# Multiplexed real-time PCR for the detection and differentiation of *Klebsiella pneumoniae* O-antigen serotypes

**DOI:** 10.1128/spectrum.00375-24

**Published:** 2024-08-08

**Authors:** Damien Slater, Kian Hutt Vater, Sushmita Sridhar, Wontae Hwang, Derek Bielawski, Sarah E. Turbett, Regina C. LaRocque, Jason B. Harris

**Affiliations:** 1 Division of Infectious Diseases, Massachusetts General Hospital, Boston, Massachusetts, USA; 2 Department of Pediatrics, Harvard Medical School, Boston, Massachusetts, USA; 3 Department of Pathology, Massachusetts General Hospital, Boston, Massachusetts, USA; 4 Department of Medicine, Massachusetts General Hospital, Boston, Massachusetts, USA; 5 Harvard Medical School, Boston, Massachusetts, USA; Michigan State University, East Lansing, Michigan, USA

**Keywords:** *Klebsiella pneumoniae*, O-antigen, serotyping, real-time PCR

## Abstract

*Klebsiella pneumoniae* has emerged as a global health threat due to its role in the spread of antimicrobial resistance and because it is a frequent cause of hospital-acquired infections and neonatal sepsis. Capsular and lipopolysaccharide (LPS) O-antigen polysaccharide surface antigens are major immunogens that are useful for strain classification and are candidates for vaccine development. We have developed real-time PCR reagents for molecular serotyping, subtyping, and quantitation of the most prevalent LPS O-antigen types (i.e., O1, O2, O3, and O5) of *Klebsiella pneumoniae*. We describe two applications for this O-typing assay: for screening culture isolates and for direct typing of *Klebsiella pneumoniae* present in stool samples. We find 100% concordance between the results of the O-typing assay and whole-genome sequencing of 81 culture isolates, and >90% agreement in O-typing performed directly on specimens of human stool, with disagreement arising primarily from a lack of sensitivity of the culture-based comparator method. Additionally, we find evidence for mixed O-type populations at varying levels of abundance in direct tests of stool from a hospitalized patient population. Taken together, these results demonstrate that this novel O-typing assay can be a useful tool for *K. pneumoniae* epidemiologic and vaccine studies.

**IMPORTANCE:**

*Klebsiella pneumoniae* is an important opportunistic pathogen. The gastrointestinal (GI) tract is the primary reservoir of *K. pneumoniae* in humans, and GI carriage is believed to be a prerequisite for invasive infection. Knowledge about the dynamics and duration of GI carriage has been hampered by the lack of tools suitable for detection and strain discrimination. Real-time PCR is particularly suited to the higher-throughput workflows used in population-based studies, which are needed to improve our understanding of carriage dynamics and the factors influencing *K. pneumoniae* colonization.

## INTRODUCTION

*Klebsiella pneumoniae* (Kp) is a gram-negative, non-motile, encapsulated bacterium of the Enterobacteriaceae family that is found in many animal and environmental reservoirs. In humans, Kp colonizes the oropharynx, gastrointestinal (GI) tract, and vagina (1) and in the elderly, immunocompromised, and those with underlying medical conditions, it can cause opportunistic infections, including pneumonia, urinary tract infections, and bacteremia (2, 3). Endogenous dissemination from the GI tract is considered a major source of hospital-acquired Kp infections, which account for up to 10% of all nosocomial bacterial infections in the US (3, 4). Kp has also emerged as a dominant cause of neonatal sepsis, particularly in low-income settings (5, 6).

The emergence of hypervirulent (hv) and multi-drug-resistant strains of Kp over the past decades is a great concern. For hvKp, its hallmark is a significant increase in capsule production; these hypermucoid strains are more resistant to several components of the host immune response (7) and can cause invasive infections in immunocompetent individuals, with significant morbidity and mortality (8). Antibiotic resistance is an even larger issue within this species. Kp is intrinsically resistant to select narrow-spectrum beta-lactams and readily acquires and disseminates antibiotic resistance genes for major classes of antibiotics (i.e., aminoglycosides, quinolones, tetracyclines, carbapenems, polymyxins, and extended-spectrum beta-lactams) (9). With reports of strains that exhibit resistance to nearly all antibiotics, the World Health Organization has placed the organism on its priority list of pathogens needing new prevention and therapeutic strategies (10).

Several Kp vaccines are under development (11, 12). Most target the major bacterial surface antigens, capsule (K-antigen) and lipopolysaccharide O-specific polysaccharide (O-antigen). While the K-antigens are a highly diverse group of more than 80 defined serotypes, O-antigens are less heterogeneous, with approximately 80%–90% of characterized clinical isolates belonging to four serotypes: O1, O2, O3, and O5 (13–15). O1 and O2 are closely related serotypes that share a repeating galactan structure, encoded by the *rfb* operon. O1 serotypes differ from O2 serotypes by the presence of an additional set of glycosyltransferase genes (*wbbYZ*) unlinked to the *rfb* locus that cap terminal residues of the O2-polysaccharide chain (16). Additional antigenic diversity has been described for O1/O2 and O3 types; subtype classifications for O1/O2 (e.g., O2a, O2ac, O2afg, O2aeh) differ in their side chain or repeating unit structures (17), and subtype classifications for O3 (O3a, O3b, O3c) differ in the number of mannose residues (18). Genetic determinants for the various O1/O2 subtypes are also classified by a second system (e.g., O1/O2v1, O1/O2v2, and O1/O2v3) (15).

Historically, antibody-based O-serotyping methods were considered gold standard but have gradually been replaced by molecular typing methods. Bacterial whole-genome sequencing (WGS) is commonly used, and an endpoint PCR test method has also been adopted (19). Real-time PCR offers advantages over WGS and antibody-based methods due to its improved sensitivity, cost, turn-around time, quantitative output, and ability to integrate into higher-throughput systems. For example, an O-typing real-time PCR assay could be used for direct testing of stool samples, as a stand-alone test, or in combination with other methods for population-based studies to evaluate the prevalence and carriage dynamics of Kp serotypes in different settings. The aim of this study was to develop a real-time PCR assay to detect the most prevalent Kp O-types and subtypes from Kp isolates as well as directly from stool specimens. Here, we characterize the performance of the O-typing multiplexed real-time PCR sets.

## MATERIALS AND METHODS

### Bacterial stocks

Bacterial isolates used in this study were obtained from commercial, academic, and internal sources. A detailed list is provided in Table S1. Blood culture isolates of Kp were collected by the Massachusetts General Hospital (MGH) Clinical Microbiology Laboratory as part of routine clinical care. Bacterial isolates were identified using the laboratory’s standard operating procedures and confirmed by WGS as outlined below.

### Stool specimens

De-identified discarded stool specimens were obtained from the MGH Clinical Microbiology Laboratory under an IRB-approved protocol. Specimens were submitted for *Clostridium difficile* testing and were stored and refrigerated in their original specimen collection containers for 2–4 days prior to release for use in this study.

### *Klebsiella pneumoniae* isolation from stool

Kp was isolated from stool specimens following a procedure based on the enrichment culture method described by Huynh et al. (5). Stool samples were first diluted to a concentration of 200 mg/mL in 15% glycerol in phosphate-buffered saline (PBS-G). Samples were homogenized and inoculated into Luria-Bertani broth (BD-Difco, Sparks, MD) supplemented with ampicillin. Enrichment cultures were incubated 18 hours at 37°C prior to plating 100 μL onto Simmons Citrate Agar (Sigma-Aldrich, St. Louis, MO) with inositol (SCA-I) and then incubated 48 hours at 37°C. For agar plates with heavy growth, where individual colonies could not be isolated, a streak of the bacterial lawn was subcultured on SCA-I for 48 hours. Ten colonies suspected to be Kp, based on colony morphology, were selected for each sample, and a pooled stock was created to reduce the scale of downstream testing. *K. pneumoniae* identity was confirmed by Kp quantitative PCR (Kp qPCR; [20]).

### Nucleic acid extraction and quantitation

Bacterial genomic DNA was prepared using a QIAamp DNA Mini Kit (Qiagen, Germantown, MD) and quantitated using a Qubit 4 Fluorometer (ThermoFisher Scientific, Waltham, MA). Estimations of genome copy number per unit mass for analytical studies were calculated using the reported median total genome size from NCBI genome (i.e., 5.59628 Mb for *Klebsiella pneumoniae*).

Stool specimen DNA was extracted using a MagMAX Microbiome Ultra Kit (Life Technologies, Carlsbad, CA) with the KingFisher Flex System (ThermoFisher Scientific, Waltham, MA). Four-hundred microliters of the homogenized stool (in PBS-G) were extracted and eluted using MagMAX kit elution buffer.

### PCR

For real-time PCR, primers and probes for the O-typing sets were obtained from Integrated DNA Technologies (Coralville, IA). Primers and MGB probes for the Kp qPCR set (20) were purchased from Thermo Fisher Scientific (Waltham, MA). Final primer and probe concentrations for all sets were 200 nM. Five microliters of template were tested in a 20-μL PCR using 2× iQ Multiplex Powermix (Bio-Rad, Hercules, CA). Positive and negative controls were included on each run. The PCR conditions used for all sets were a single 2-minute 95°C hot-start activation step, followed by 40 cycles of 95°C for 10 seconds, and 60°C for 1 minute, using an ABI 7500 Fast instrument (Thermo Fisher Scientific, Waltham, MA). Automatic baseline detection was used for all sets except the O3 set, where a manual-defined baseline of cycle 3–15 was used. Cycle threshold values of 20,000 were used for all O sets, except O5, which was set to 10,000. A cycle threshold of 50,000 was used for both the *fiu* and 23S sets of the Kp qPCR.

Kp O-typing endpoint PCR was performed following the method of Fang et al. (19). PCR primers were obtained from Integrated DNA Technologies (Coralville, IA).

### Analytical validation

Linearity for the two multiplex panels was tested using 5 × 10-fold serial dilutions of Kp DNA from well-characterized isolates The lower limit of detection (LoD) for O-types was determined by testing twofold dilutions of DNA from 10 to 1.25 copies/reaction in replicates of 20. The LoD was assigned to the lowest analyte concentration with an observed detection rate ≥95% for each set. Assay precision was tested with quantitated stocks of cultured Kp spiked into analyte-negative stool matrix. Stool matrix was derived from four individual stool samples and prepared in PBS-G. Spiked stool samples were prepared at three analyte concentrations (i.e., 10–20× LoD, 2–3× LoD, and <1× LoD). Samples were extracted and tested in duplicate over a 10-day period by a single individual. Specificity was tested against a panel of enteric bacteria that included other *Klebsiella* species and closely related Enterobacteriaceae*,* at a concentration of ≥10^6^ genome copies per reaction.

### Clinical validation

Two method comparison studies were conducted to evaluate the clinical performance of the assay. The first study compared O-antigen typing assignments by the real-time PCR assay to those made by Kaptive (15) from whole-genome sequence data from a panel of 81 isolates obtained from *K. pneumoniae* bacteremia cases at Massachusetts General Hospital collected during the period of July 2021 to February 2022 (21).

The second study compared O-antigen typing assignments from real-time PCR made directly from stool samples to those from an established endpoint PCR typing assay (19) made on a pool of 10 bacterial isolates from Kp enrichment culture that were confirmed positive by Kp qPCR. Test specificity was boosted by the inclusion of an additional real-time PCR assay for detection of Kp (20) in the test method, which measures the relative abundance of Kp by comparing the C*_T_* values for Kp to total bacterial 23S rRNA. The final method uses Kp qPCR assay to identify samples positive for Kp*,* which are then reflexed to the O-typing real-time PCR assay for serotyping assignment. Method comparison was performed on 132 stool sample remainders that had been submitted to the MGH Clinical Microbiology Laboratory for *C. difficile* testing.

### Whole-genome sequencing

Genomic DNA from Kp isolates was sent to the Vanderbilt University Medical Center core facility (Vantage, Nashville, TN) for whole-genome sequencing. The Twist Biosciences (San Francisco, CA) NGS Library Prep Kit was used for library preparation, and 150-base paired-end reads were collected on an Illumina NovaSeq6000 system (San Diego, CA). An average of 10 million reads were obtained for each sequenced isolate. Raw reads were subjected to adapter trimming with Trimmomatic (v0.39) and quality control by FastQC (v0.11.9) (22) and then aligned to reference strain NTUH-K2044 (accession PRJDA21069) (23) using Pilon (v1.24) (24). Adapter-trimmed sequences were assembled using SPAdes (v3.15.3) (25) using default parameters. Assembly files were then used to identify O-, K-, and ST-types with Kleborate v2.0.0 (15, 26). Whole-genome sequences are available in the Sequence Read Archive BioProject PRJNA978102 (21).

### Statistics

GraphPad Prism (San Diego, CA) was used for statistical analyses. The ΔΔC*_T_* method (20) was used for determining the relative abundance Kp and Kp O-type(s) in stool samples. Linear regression was used to evaluate the linearity of detection and to correlate C*_T_* values for *fiu* and O-antigen sets. The Mann-Whitney non-parametric *t*-test was used for comparisons between sample C*_T_* values and for differences in Kp relative abundance.

## RESULTS

### O-typing assay design

Candidate target regions for the O-antigen typing sets were identified by screening the *rfb* locus of members of the Kp species complex for well-conserved 200-base pair regions. Multiple sequence alignments were made from a non-redundant set of sequences that represented all variation in each inclusion group (accession IDs provided in Table S2), then these regions were compared to those of other Kp O-types as well as sequences from other *Klebsiella* and closely related Enterobacteriaceae species for specificity. *In silico* analyses of primer probe candidate sets were performed with Visual OMP to evaluate the performance of all sequences within the inclusion and exclusion groups, in both individual and multiplexed format. A Visual OMP %bound score of <90% was used as a cutoff for determining if additional primers or degenerate bases were needed to cover all variation within the inclusion set. Degenerate bases were used if sequence variation was limited to one or two bases in the primer sequence. Additional primers were used for cases in which there were more than two variant base positions in the primer sequence, as with the O5 set forward and reverse primers.

NCBI BLAST searches of the nr/nt collection with each of the primers in the multiplex were used to screen for potential cross-reactants. Sequences detected in the BLAST screen were flagged for further analysis with Visual OMP if binding of one or more of the primers in the multiplex could result in a PCR product of ≤2 kb. The only notable cross-reactivity predicted for this screen was for *E. coli* strains belonging to the O8- and O9/9a-antigen types. However, cross-reactivity for these strains was expected, having been reported previously for Kp O5 and O3 genotypes with *E. coli* O8 and O9/9a types, respectively (16). The O5 set was designed with this in mind, with primer placement creating 3′-terminal base mismatches that were predicted to be non-extensible for *E. coli* O8 sequences but not Kp O5 strains. No design strategy was identified to improve the specificity of the Kp O3-antigen set, so cross-reactivity with some strains from the *E. coli* O9/9a genotype is likely. This predicted cross-reactivity with *E. coli* was limited to the O3 set and was not observed for the O3b-antigen set. O-antigens excluded from the PCR design (e.g., O2ac, O4, O12, and OL101-104) were omitted due to either predicted specificity issues (i.e., high-sequence identity within the *rfb* locus of other Enterobacteriaceae) or low prevalence among circulating strains.

**Fig 1:**
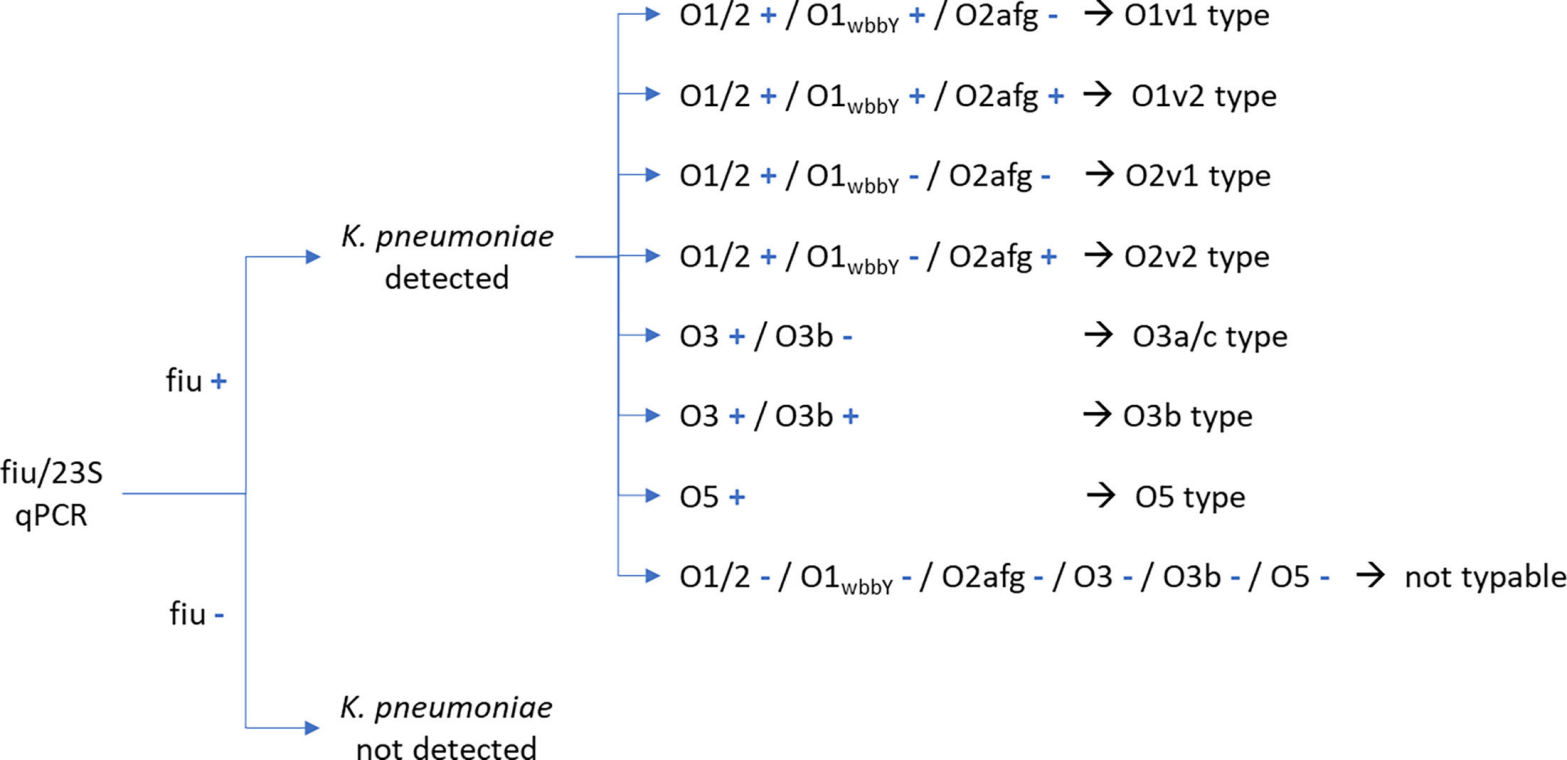
Real-time PCR result calling scheme.

The final design format combined six target regions in two multiplexed sets and allowed typing of O1, O2, O3, and O5 strains as well as subtyping of O1 (O1v1, O1v2), O2 (O2v1, O2v2), and O3 (O3a, O3b). For assay result reporting, the remaining O-types (e.g., O4, O12, and OL101-104) were grouped into a “non-typable *K. pneumoniae*” classification. An overview of target regions, multiplex format, and oligonucleotide sequences is provided in Table 1. The call assignment method for O-typing/subtyping is shown in Fig. 1.

**TABLE 1:** Primer and probe sequences for O-typing multiplex real-time PCR sets

Set	O-Ag type	Gene	Oligonucleotide	Sequence (5*'*−3*'*)	Final conc. (nM)	Amplicon length (nt)
1	O1 and O2	wzm	O1/2_F	CATTCATYGCTAAYGCTCAAATTA	200	120
			O1/2_R	ARACRACAATAACCGGGATGGT		
			O1/2_P	/56-FAM/CCGGTCCGT/ZEN/GATTCCGCTAAGTAA/3IABkFQ		
	O1	wbbY	O1_F	TCGATGAGATTCAAATAACGATGAGAA	200	89
			O1_R	AGCGTGTGTATAACTCACTTCGAA		
			O1_P	/5SUN/ACTTCCATC/ZEN/ATCAACCAAGATGACCTCA /3IABkFQ/		
	O3b	wbdD	O3b_F	ACTCACCTCCCTGATTAATATTATGG	200	93
			O3b_R	GGCTGTACRTTAGTAGTAACGTCTT		
			O3b_P	/5TexRd-XN/TTTGAGAAGCGTGAGTATCCGCCA/3IAbRQSp/		
	O5	wzt	O5_F1	AGGACGATATTCCTGAGCTAGTC	200	101
			O5_F2	AGGCTGATATCCCTGAGCTCGTC		
			O5_F3	AGGACGATATTCCTGAACTTGTCG		
			O5_R1	GGTGAGAGTCTGTTTGAGATGATG		
			O5_R2	GGTGAGTGTTTGTTTGAGATGATG		
			O5_R3	GGTGAGTGTCTGTTTAAGATGATG		
			O5_P	/5Cy5/TCGGATATA/TAO/TGATCAAGGATCGACTAGGG/3IAbRQSp/		
2	O1v2 and O2v2 (O2afg)	gmlC	O2afg_F1	TAGTCATAGCGATCGGGATAGGTTC	200	95
			O2afg_F2	TAGTCATAACGATCTGGATAGGTTC		
			O2afg_F3	TAGTCATAGTGATCGGGATAGGTTC		
			O2afg_R	AATAAGTTCTAAGGCCACTAA YGAG		
			O2afg_P	/56-FAM/AAAGAGAAT/ZEN/TGGGCAGCATTCCGC/3IABkFQ/		
	O3	wzm	O3_F	AACGATCAGCACCGGRATG	200	112
			O3_R	CTCGGCGTTCATTAACTTYCT		
			O3_P	/5TEX615/AAATCATGCCYGGGAAATTGCCC/3IAbRQSp/		

### Analytical performance

The assay was determined to be linear for the full range of input concentrations tested (5 × 10^5^–5 genome copies per reaction) for each O-antigen type covered by the assay. PCR amplification efficiencies for the different targets ranged from 86.6% to 99.7% with *R*^2^ values >0.98 (Table 2). The limit of detection was five genome copies per PCR for each O-type, except for O5, which was 10 copies per PCR. Factoring in the extraction process, these translate to LoDs in the range of 2.5–5 × 10^3^ colony forming units per gram of stool.

**TABLE 2:** Analytical performance summary

O-type	Strain	Target	PCR efficiency (%)	Linearity	Precision (%CV)		LoD
					Intra-assay	Inter-assay	(genomes/PCR)
O1	MGH KPN003	O1/2	88.1	0.992	0.7–2.9	1.7–3.6	5
		O1wbbY	92.9	0.992	0.4–2.1	1.2–2.2	
O2a	MGH KPN006	O1/2	89.6	0.997	1.0–3.1	1.1–2.7	5
O2afg	MGH KPN030	O1/2	89.5	0.997	1.2–3.0	2.0–7.4	5
		O2afg	99.7	0.998	0.9–2.4	1.7–2.8	
O3/O3a	MGH KPN047	O3	89.7	0.996	1.5–2.4	1.7–3.9	5
O3b	MGH KPN001	O3	86.6	0.982	2.1–3.8	2.3–3.0	5
		O3b	99.8	0.994	1.1–3.6	2.0–3.0	
O5	MGH KPN049	O5	97.3	0.996	1.2–2.5	1.5–2.5	10

Specificity was tested against a panel of enteric bacteria that included other *Klebsiella* species and closely related Enterobacteriaceae (Table S1), at a concentration of ≥10^6^ genome copies per reaction. Each organism was tested with the O-typing sets as well as a second previously validated assay for detection of Kp (20). The panel included 42 *E. coli* strains and isolates representing 29 O genotypes. Cross-reactivity of the O3 set with *E. coli* O9/9a-types that was predicted in our *in silico* analyses was confirmed, for all seven of the *E. coli* O9/9a genotypes tested. However, no cross-reactivity was observed for any other of the 29 *E. coli* O-types tested, including the closely related O8 type. The O5 set was found to cross-react with one of the two strains of *Klebsiella aerogenes* tested. No cross-reactivity was observed for any of the bacterial species tested with the Kp qPCR set.

To determine how C*_T_* values for the Kp qPCR (20) and the O-typing real-time PCR assay correlated, we tested serial dilutions of Kp DNA prepared in a background of stool DNA from a pool of four Kp-negative individuals. The three tested strains encompass all assay targets; all showed very high levels of correlation between *fiu* and O-typing C*_T_* values, *r* ≥0.98 (Fig. S1a). Since C*_T_* values for the assays were closely correlated, we used linear regression equations to estimate that O-typing C*_T_* values should be within 2.2 cycles of the *fiu* C*_T_* value, for *fiu* C*_T_* values between 20 and 35 (Fig. S1b). Consequently, we include a comparison of *C*_*T*_ values for the two assays as part of the result calling process. Those samples where the C*_T_* difference between *fiu* and O-type exceeds three cycles are flagged for further scrutiny. In addition, to further improve confidence in O-typing assignments, C*_T_* cutoffs for the two assays were established for samples with near-LoD Kp levels (Table S3).

### Clinical performance: O-antigen typing of *K. pneumoniae* isolates

Accuracy for the O-typing real-time PCR assay was assessed using a panel of 81 isolates from *K. pneumoniae* bacteremia cases at Massachusetts General Hospital, collected during the period of July 2021 to February 2022 (21). O-antigen assignments for PCR were compared to those made by Kaptive (15) from whole-genome sequence data. Positive agreement for the two methods was 100% (Table 3). Serotyping assignments were made for more than 85% (69/81) of the isolates tested, and all O-types and sub-types included in the assay design were represented in the population. The most prevalent O-types observed were O1v2 (17/81, 20.9%) and O3b (16/81, 19.8%).

**TABLE 3:** Method comparison results for O-typing real-time PCR with whole-genome sequencing for Kp isolates

O-type	*N*	% Of total tested	% Agreement with WGS
O1v1	11	13.6%	100
O1v2	17	21.0%	100
O2v1	5	6.2%	100
O2v2	8	9.9%	100
O3/O3a	4	4.9%	100
O3b	16	19.8%	100
O5	8	9.9%	100
Non-O1, O2, O3, O5^*a*^	12	14.8%	100
Total	81	100.0%	100

^*a*^ WGS call for isolates: O4 (*n* = 4), O12 (*n* = 2), OL101 (*n* = 3), OL103 (*n* = 2), OL104 (*n* = 1).

### Clinical performance: direct O-typing method comparison for stool

**Fig 2:**
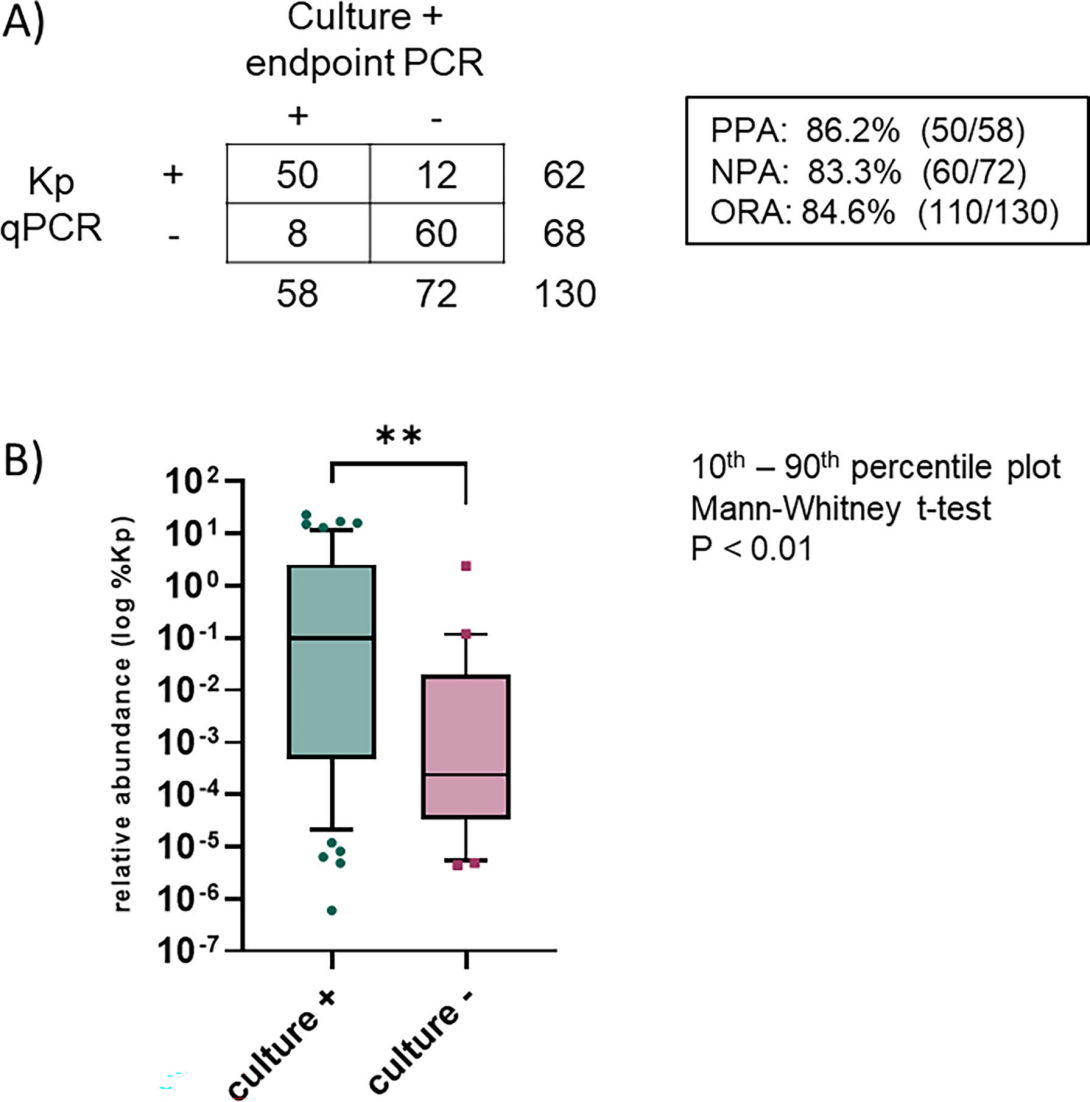
Method comparison results for Kp qPCR with culture for stool samples. (**A**) Comparison of results for the detection of *K. pneumoniae* by Kp qPCR and culture. PPA, positive percent agreement; NPA, negative percent agreement; ORA, overall rate of agreement. (**B**) Relative abundance of *K. pneumoniae* in culture-positive and culture-negative samples, as determined by Kp qPCR using the ΔΔC*_T_* method.

To evaluate the performance of the O-typing real-time PCR method directly on stool samples, we obtained 132 stool remainders from the MGH Clinical Microbiology Laboratory and compared results for direct O-typing real-time PCR to results from an O-typing endpoint PCR (19) of Kp isolates from enrichment culture of the same stool samples. Kp was detected in a high proportion of samples by both methods: Kp qPCR (62/130, 47.7%) and enrichment culture (58/130, 44.6%) (Fig. 2A). Two samples (2/132, 1.5%) were excluded from the analysis due to PCR inhibition that impacted detection of the 23S rRNA set. Overall agreement for Kp detection between the Kp qPCR and culture method was 84.6% (86.2% positive call agreement, 83.3% negative call agreement, Fig. 2A). The relative abundance of Kp in samples belonging to the culture-negative group was significantly lower than that observed for the culture-positive group (median values of 0.098% and 0.0002%, respectively, *P* < 0.01), which likely contributed to the difference in Kp detected with the two methods (Fig. 2B).

**Fig 3:**
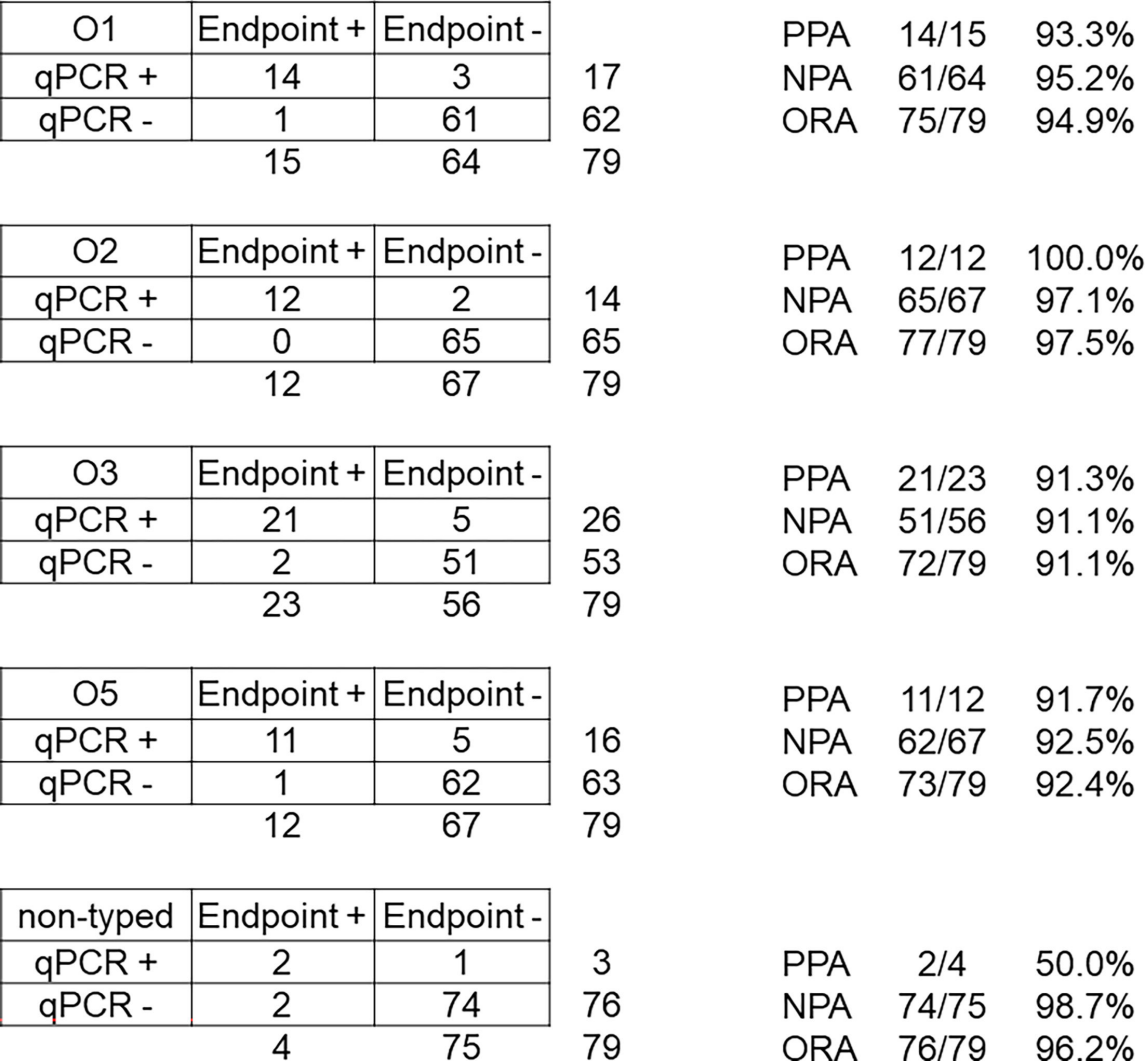
Method comparison results for O-typing real-time PCR with culture for stool samples. ORA, overall rate of agreement.

O-antigen typing calls were made in all 50 samples where Kp was detected by both PCR and culture. A total of 79 O-antigen calls were made (76 by direct real-time PCR, 66 by isolate PCR), and 60/79 (75.9%) of calls agreed for the two methods (Fig. 3). Individually, each O-antigen type had an overall method agreement of >90%: 94.9% for O1 [93.3% positive percent agreement (PPA), 95.2% positive percent agreement (NPA)]; 97.5% for O2 (100% PPA, 97.1% NPA); 91.1% for O3 (91.3% PPA, 91.1% NPA); 92.4% for O5 (91.7% PPA, 92.5% NPA); and 96.2% for non-typable calls (50% PPA, 98.7% NPA) (Fig. 3). In all cases, discrepancies could be attributed to a lack of detection, and not from O-type assignment disagreements, with 15 calls missed by culture and 4 by real-time PCR. The limited sensitivity of the culture comparator method was supported by results from our discrepancy investigation using a scrape of colonies from the SCA-I plate, where the original O-typing real-time PCR result was confirmed for most cases (Table S4).

### Stool samples with multiple O-types

**Fig 4:**
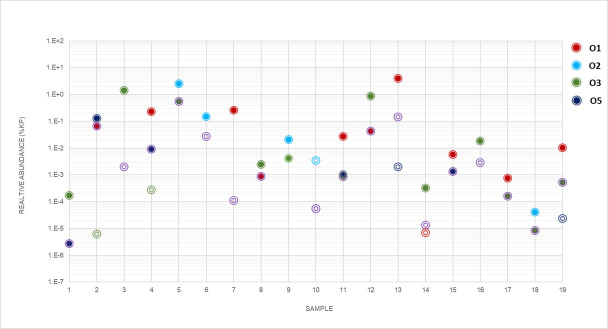
Relative abundance of *K. pneumoniae* O-types in samples with mixed populations. Relative abundance of O-types in 19 samples with mixed populations detected by real-time PCR and/or culture. Abundance of each type was calculated by the ΔΔC*_T_* method, baselining O-type C*_T_* value to bacterial 23S rRNA. O-types are represented by color (O1, red; O2, blue; O3, green; O5, purple). Filled circles denote real-time calls that were confirmed by culture, and open circles indicate calls that were not confirmed by culture.

Of the 50 stool samples where Kp was detected by both Kp PCR and culture method, 19 (38.0%) had multiple O-types detected by direct real-time PCR and 15 (30%) by isolate endpoint PCR. The relative of abundance of each O-type in samples was calculated by the ΔΔC*_T_* method using 23S C*_T_* values from the Kp qPCR assay. Distribution patterns varied with respect to the most abundant O-types in the samples as well as the relative abundance of O-types in the same sample. For some samples, O-types were present in similar concentrations, while up to 20,000-fold differences in O-types were observed in other samples (Fig. 4).

### Inferred detection of O-types not targeted by the assay

Based on reported Kp prevalence estimates (14, 15), O-types that are not covered by our assay design are expected to be encountered in up to 15% of samples. The presence of a non-typable strain was suspected if the *fiu* C*_T_* value was more than five cycles lower than any of the O-antigen C*_T_* value or if endpoint PCR tests of cultured isolates did not yield a result for any of the O1, O2, O3, or O5-typing sets. Six Kp qPCR-positive samples (6/62, 9.7%) were flagged with this C*_T_*-differential method as possibly carrying one of the Kp O-types not targeted by the assay. Discrepant testing with endpoint PCR confirmed the presence of O9 and O12 types in two of the six samples (MGHK077 and MGHK101, Table S4). Coverage of the endpoint PCR assay includes O4, O8, O9, and O12 types, but not OL101-OL104, so the remaining four non-typable strains may belong to one of those O-types.

## DISCUSSION

Here, we describe the development and validation of a real-time PCR method for genotyping the most prevalent Kp O-antigen serotypes. The assay provides a rapid, reliable method for screening culture isolates and when paired with a second real-time PCR assay for *K. pneumoniae*, provides a novel method for measuring the presence of specific Kp O-types directly from stool samples.

O-typing has traditionally been performed on culture isolates, using serological tests or whole-genome sequencing (27, 28); more recently, a metagenomic sequencing approach from enriched culture was also evaluated (29). A direct method of O-typing from stool, such as the one described here, is useful for situations when culture of viable bacteria is challenging (e.g., archived frozen samples and presence of antibiotics). It also provides an additional granularity to screening methods by measuring the relative abundance of Kp in stool, an approach whose utility has been recognized for identifying individuals at risk of infection or for identifying potential “super-spreaders” in hospital settings (20, 30–33). In our own research, we expect direct testing of stool to be useful in epidemiologic studies measuring the dynamics of serotype carriage and the factors influencing colonization persistence. We also imagine that it could be employed in future studies of vaccine effectiveness.

Evaluations of analytical performance of our assay demonstrated good sensitivity and precision for the O-serotyping sets. Tests of assay specificity confirmed the expected cross-reactivity with *E. coli* O9/9a strains, but no cross-reactivity was noted for *E. coli* O8 or any of the other 28 *E. coli* O-types tested. Cross-reactivity was also observed for the O5 set with one strain of *K. aerogenes*. However, potential false-positive assay calls from non-Kp bacteria are mitigated by the inclusion of a real-time PCR set specific for Kp in our direct test method for stool, which is supported by the previous assay validation study (20) and our observation that no cross-reactivity was observed with any in our specificity panel comprising closely related and other common enteric bacteria.

When used as a rapid screening method for Kp clinical isolates, our O-typing real-time PCR provided O-typing assignments that agreed with Kaptive calls from whole-genome sequence data for all 81 isolates tested. The prevalence of typable strains (~85%) in this study, as well as the most commonly observed types (i.e., O1 and O3), was consistent with other reports (14, 15). Likewise, results for direct O-typing from stool were generally good, with disagreements resulting mainly from a lack of sensitivity of the culture comparator method. While the overall positive agreement for typing calls for the method comparison study was ~76%, many of the Kp-positive samples had more than one O-type detected, and differences in detection of the lower abundant O-types were responsible for most of the observed discrepancies. If the comparison was restricted to only the dominant O-type for each sample (i.e., lowest C*_T_* value), the positive agreement would be 94% (47/50).

An unexpected finding in this study was the frequency with which we encountered multiple Kp O-types in clinical stool samples. Intestinal co-carriage of multiple Kp strains has been described previously (34–36), and for other Enterobacteriaceae (37, 38), but reports are limited, and it is unclear how frequently this occurs. We observed that the relative abundance of Kp strains could vary by up to several logs; this wide range may account for the limitations of previous culture-based methods in detecting multiple O-types in stool. Metagenomic sequencing approaches are now starting to be used for Kp epidemiologic studies and have the sensitivity to detect minor strains present at 0.1%–1.0% level of the abundant strain, so such an approach could provide additional insight. Nevertheless, one recent study using a metagenomic approach did not find evidence for co-colonization in their study population (28). Such differences may arise from the demographics of the study populations or possibly sample type (i.e., stool vs rectal swab). Further work is needed with larger, better-defined, and varied populations to appreciate the frequency of co-carriage of different Kp O-types.

Our direct O-typing PCR method, while useful, does have some limitations. Interpretation of results can be nuanced in cases where more than one O-type is present, including those where an O-type not targeted by the assay is present. In these cases, the primary, more abundant O-type call is typically clear, but O-type assignments for the additional strains may require more scrutiny. It is also worth noting that there may be instances of co-carriage where resolution of calls for O-types may not be possible due to common genetic elements (e.g., O2 and O1, or O3a and O3b). Finally, though we did not find any evidence for cross-reactivity with O3 or O5 sets, we recognize that co-carriage of *K. pneumoniae* and *E. coli* O9/9a and/or *K. aerogenes* strains could complicate result interpretation. The prevalence of *E. coli* O9 is not clear. One study reported *E. coli* O9 strains were present in between 0.6% and 2.9% of *E. coli* bacteremia cases across varying geographical locations (39), but this may not reflect carriage rates in the general population.

In summary, we report the development of novel serotyping tools for the most prevalent *K. pneumoniae* LPS O-antigens and demonstrate their utility for rapid screening of culture isolates and for direct testing of stool samples. Integrating this method into high-throughput workflows can provide value for studies examining *K. pneumoniae* carriage dynamics in healthy and hospitalized populations and possibly in future intervention effectiveness studies.

## Data Availability

Sequencing data can be found at Sequence Read Archive with Bioproject no. PRJNA978102.

## SUPPLEMENTAL MATERIAL

Supplemental material spectrum.00375-24-s0001.docx Tables S1 to S4; Fig. S1.

ASM does not own the copyrights to Supplemental Material that may be linked to, or accessed through, an article. The authors have granted ASM a non-exclusive, world-wide license to publish the Supplemental Material files. Please contact the corresponding author directly for reuse.
