# Supplementary material for: Multiplexed real-time PCR for the detection and differentiation of Klebsiella pneumoniae O-antigen serotypes
Source: Microbiol Spectr. 2024 Aug 8;12(9):e00375-24. doi: 10.1128/spectrum.00375-24 (PMC11371267; doi:10.1128/spectrum.00375-24)
Supplement: Supplemental material — Tables S1 to S4; Fig. S1. [file spectrum.00375-24-s0001.docx]

**Table S1**. Specificity Panel Results

| **Organism** | **Strain/Source** | **O-type** | **23S**  **C*_T_*** | **Kpn_fiu**  **C*_T_*** | **O1/O2 C*_T_*** | **O1wbbY C*_T_*** | **O2afg C*_T_*** | **O3**  **C*_T_*** | **O3b**  **C*_T_*** | **O5 C*_T_*** |
| --- | --- | --- | --- | --- | --- | --- | --- | --- | --- | --- |
| *Salmonella enterica subsp. enterica serovar Typhi* | CT18 | ND | 18.0 | ND | ND | ND | ND | ND | ND | ND |
| *Salmonella enterica subsp. enterica serovar Typhimurium* | ATCC 14028 | ND | 17.6 | ND | ND | ND | ND | ND | ND | ND |
| *Shigella flexneri 2a* | isolate from Edward Ryan, MGH | ND | 20.4 | ND | ND | ND | ND | ND | ND | ND |
| *Shigella sonnei* | isolate from Edward Ryan, MGH | ND | 22.8 | ND | ND | ND | ND | ND | ND | ND |
| *Shigella boydii* | isolate from Edward Ryan, MGH | ND | 22.1 | ND | ND | ND | ND | ND | ND | ND |
| *Klebsiella oxytoca* | BEI MIT-5248 | ND | 13.2 | ND | ND | ND | ND | ND | ND | ND |
| *Klebsiella oxytoca* | BEI MIT-5249 | ND | 16.5 | ND | ND | ND | ND | ND | ND | ND |
| *Klebsiella oxytoca* | BEI MIT-5250 | ND | 17.0 | ND | ND | ND | ND | ND | ND | ND |
| *Enterobacter cloacae* | BEI 102 | ND | 17.5 | ND | ND | ND | ND | ND | ND | ND |
| *Klebsiella aerogenes* | BEI CRE UCI-15 | ND | 13.7 | ND | ND | ND | ND | ND | ND | 16.9 |
| *Pseudomonas aeruginosa* | BEI PA14 | ND | 18.1 | ND | ND | ND | ND | ND | ND | ND |
| *Yersiina entercolitica* | BEI NR-206 | ND | 14.0 | ND | ND | ND | ND | ND | ND | ND |
| *Campylobacter coli* | BEI 296 JV20 | ND | 25.5 | ND | ND | ND | ND | ND | ND | ND |
| *Campylobacter jejuni* | ATCC 33560 | ND | 26.8 | ND | ND | ND | ND | ND | ND | ND |
| *Campylobacter upsalensis* | BEI HM-297 JV21 | ND | 26.5 | ND | ND | ND | ND | ND | ND | ND |
| *Citrobacter freundii* | GTEN isolate | ND | 17.5 | ND | ND | ND | ND | ND | ND | ND |
| *Citrobacter freundii* | GTEN isolate | ND | 17.3 | ND | ND | ND | ND | ND | ND | ND |
| *Klebsiella aerogenes* | GTEN isolate | ND | 15.5 | ND | ND | ND | ND | ND | ND | ND |
| *Escherichia hermannii* | GTEN isolate | ND | 17.4 | ND | ND | ND | ND | ND | ND | ND |
| *Citrobacter youngae* | GTEN isolate | ND | 17.9 | ND | ND | ND | ND | ND | ND | ND |
| *Enterobacter cancerogenus* | GTEN isolate | ND | 18.1 | ND | ND | ND | ND | ND | ND | ND |
| *Enterobacter kobei* | GTEN isolate | ND | 18.0 | ND | ND | ND | ND | ND | ND | ND |
| *Escherichia coli* | GTEN_001 isolate | O001 | 17.1 | ND | ND | ND | ND | ND | ND | ND |
| *Escherichia coli* | GTEN_070 isolate | O003 | 16.7 | ND | ND | ND | ND | ND | ND | ND |
| *Escherichia coli* | GTEN_072 isolate | O005 | 17.7 | ND | ND | ND | ND | ND | ND | ND |
| *Escherichia coli* | GTEN_053 isolate | O007 | 16.0 | ND | ND | ND | ND | ND | ND | ND |
| *Escherichia coli* | GTEN_ 025 isolate | O008 | 18.1 | ND | ND | ND | ND | ND | ND | ND |
| *Escherichia coli* | GTEN_ 105 isolate | O008 | 17.0 | ND | ND | ND | ND | ND | ND | ND |
| *Escherichia coli* | GTEN_016 isolate | O008 | 16.7 | ND | ND | ND | ND | ND | ND | ND |
| *Escherichia coli* | GTEN_035 isolate | O008 | 17.2 | ND | ND | ND | ND | ND | ND | ND |
| *Escherichia coli* | GTEN_106 isolate | O008 | 16.9 | ND | ND | ND | ND | ND | ND | ND |
| *Escherichia coli* | GTEN_112 isolate | O008 | 16.3 | ND | ND | ND | ND | ND | ND | ND |
| *Escherichia coli* | GTEN_121 isolate | O008 | 16.1 | ND | ND | ND | ND | ND | ND | ND |
| *Escherichia coli* | GTEN_ 140 isolate | O009 | 17.8 | ND | ND | ND | ND | 24.5 | ND | ND |
| *Escherichia coli* | GTEN_141 isolate | O009 | 17.2 | ND | ND | ND | ND | 24.5 | ND | ND |
| *Escherichia coli* | GTEN_ 024 isolate | O009a | 19.6 | ND | ND | ND | ND | 25.7 | ND | ND |
| *Escherichia coli* | GTEN_037 isolate | O009a | 18.6 | ND | ND | ND | ND | 16.4 | ND | ND |
| *Escherichia coli* | GTEN_051 isolate | O009a | 15.2 | ND | ND | ND | ND | 19.2 | ND | ND |
| *Escherichia coli* | GTEN_052 isolate | O009a | 17.2 | ND | ND | ND | ND | 18.2 | ND | ND |
| *Escherichia coli* | GTEN_273 isolate | O009a | 18.9 | ND | ND | ND | ND | 16.2 | ND | ND |
| *Escherichia coli* | GTEN_058 isolate | O011 | 16.9 | ND | ND | ND | ND | ND | ND | ND |
| *Escherichia coli* | GTEN_004 isolate | O015 | 16.2 | ND | ND | ND | ND | ND | ND | ND |
| *Escherichia coli* | GTEN_007 isolate | O016 | 20.2 | ND | ND | ND | ND | ND | ND | ND |
| *Escherichia coli* | GTEN_002 isolate | O025 | 20.7 | ND | ND | ND | ND | ND | ND | ND |
| *Escherichia coli* | GTEN_327 isolate | O030 | 16.7 | ND | ND | ND | ND | ND | ND | ND |
| *Escherichia coli* | GTEN_040 isolate | O045 | 16.2 | ND | ND | ND | ND | ND | ND | ND |
| *Escherichia coli* | GTEN_068 isolate | O048 | 16.9 | ND | ND | ND | ND | ND | ND | ND |
| *Escherichia coli* | GTEN_ 298 isolate | O060 | 17.6 | ND | ND | ND | ND | ND | ND | ND |
| *Escherichia coli* | GTEN_110 isolate | O064 | 16.4 | ND | ND | ND | ND | ND | ND | ND |
| *Escherichia coli* | GTEN_015 isolate | O071 | 17.2 | ND | ND | ND | ND | ND | ND | ND |
| *Escherichia coli* | GTEN_126 isolate | O075 | 16.4 | ND | ND | ND | ND | ND | ND | ND |
| *Escherichia coli* | GTEN_126 isolate | O075 | 17.5 | ND | ND | ND | ND | ND | ND | ND |
| *Escherichia coli* | GTEN_009 isolate | O086 | 21.0 | ND | ND | ND | ND | ND | ND | ND |
| *Escherichia coli* | GTEN_ 050 isolate | O101 | 17.8 | ND | ND | ND | ND | ND | ND | ND |
| *Escherichia coli* | GTEN_064 isolate | O102 | 16.4 | ND | ND | ND | ND | ND | ND | ND |
| *Escherichia coli* | GTEN_299 isolate | O105 | 17.1 | ND | ND | ND | ND | ND | ND | ND |
| *Escherichia coli* | GTEN_438 isolate | O107 | 17.2 | ND | ND | ND | ND | ND | ND | ND |
| *Escherichia coli* | GTEN_005 isolate | O125ab | 19.4 | ND | ND | ND | ND | ND | ND | ND |
| *Escherichia coli* | GTEN_202 isolate | O138 | 16.4 | ND | ND | ND | ND | ND | ND | ND |
| *Escherichia coli* | GTEN_175 isolate | O145 | 16.7 | ND | ND | ND | ND | ND | ND | ND |
| *Escherichia coli* | GTEN_474 isolate | O149 | 16.1 | ND | ND | ND | ND | ND | ND | ND |
| *Escherichia coli* | GTEN_003 isolate | O152 | 20.1 | ND | ND | ND | ND | ND | ND | ND |
| *Escherichia coli* | GTEN_442 isolate | O154 | 16.7 | ND | ND | ND | ND | ND | ND | ND |
| *Escherichia coli* | BEI NR-4, ETEC | O178 | 17.8 | ND | ND | ND | ND | ND | ND | ND |
| ND: not detected |  |  |  |  |  |  |  |  |  |  |
| GTEN: Global Travelers Epidemiology Network U.S. Travelers Study |  |  |  |  |  |  |  |  |  |  |
| BEI: Biodefense and Emerging Infections Research Resources Repository, Manassas, VA | |  |  |  |  |  |  |  |  |  |
|  |  |  |  |  |  |  |  |  |  |  |
| ***Klebsiella pneumoniae* isolates for Reactivity Panel** | **Kleborate/Kaptive result** | | | | | | | **O-typing PCR  result** |  |  |
|  | **Species** | **ST** | **K locus** | **K type** | **O locus** | **O type** | **O locus confid.** |  |  |  |
| KPN0001; MGH sepsis isolate | *Klebsiella pneumoniae* | ST37 | KL14 | K14 | O3b | O3b | Very high | O3b |  |  |
| KPN0002; MGH sepsis isolate | *Klebsiella pneumoniae* | ST353 | KL110 | unknown (KL110) | O3b | O3b | Good | O3b |  |  |
| KPN0003; MGH sepsis isolate | *Klebsiella pneumoniae* | ST13 | KL3 | K3 | O1/O2v2 | O1 | Very high | O1v2 |  |  |
| KPN0004; MGH sepsis isolate | *Klebsiella pneumoniae* | ST1609 | KL38 | K38 | O12 | O12 | Very high | Non-O1, O2, O3, O5 |  |  |
| KPN0005; MGH sepsis isolate | *Klebsiella pneumoniae* | ST1838 | KL14 | K14 | O3b | O3b | Very high | O3b |  |  |
| KPN0006; MGH sepsis isolate | *Klebsiella pneumoniae* | ST1380 | KL31 | K31 | O1/O2v1 | O2a | Very high | O2v1 |  |  |
| KPN0007; MGH sepsis isolate | *Klebsiella pneumoniae* | ST252 | KL81 | K81 | O1/O2v2 | O1 | Very high | O1v2 |  |  |
| KPN0008; MGH sepsis isolate | *Klebsiella pneumoniae* | ST45-1LV | KL5 | K5 | O3b | O3b | Good | O3b |  |  |
| KPN0009; MGH sepsis isolate | *Klebsiella pneumoniae* | ST2623 | KL52 | K52 | OL103 | unknown (OL103) | High | Non-O1, O2, O3, O5 |  |  |
| KPN0010; MGH sepsis isolate | *Klebsiella pneumoniae* | ST29 | KL19 | K19 | O1/O2v2 | O1 | Perfect | O1v2 |  |  |
| KPN0011; MGH sepsis isolate | *Klebsiella pneumoniae* | ST37 | KL15 | K15 | O4 | O4 | Very high | Non-O1, O2, O3, O5 |  |  |
| KPN0012; MGH sepsis isolate | *Klebsiella pneumoniae* | ST20 | KL28 | K28 | O1/O2v2 | O2afg | Very high | O2v2 |  |  |
| KPN0013; MGH sepsis isolate | *Klebsiella pneumoniae* | ST461 | KL2 | K2 | O1/O2v2 | O1 | Very high | O1v2 |  |  |
| KPN0014; MGH sepsis isolate | *Klebsiella pneumoniae* | ST5142 | KL51 | K51 | O12 | O12 | Very high | Non-O1, O2, O3, O5 |  |  |
| KPN0015; MGH sepsis isolate | *Klebsiella pneumoniae* | ST920-2LV | KL56 | K56 | OL103 | unknown (OL103) | High | Non-O1, O2, O3, O5 |  |  |
| KPN0016; MGH sepsis isolate | *Klebsiella variicola subsp. variicola* | ST1582 | KL64 | K64 | O5 | O5 | Very high | O5 |  |  |
| KPN0017; MGH sepsis isolate | *Klebsiella pneumoniae* | ST219 | KL114 | unknown (KL114) | O1/O2v1 | O1 | Very high | O1v1 |  |  |
| KPN0018; MGH sepsis isolate | *Klebsiella pneumoniae* | ST22 | KL9 | K9 | O1/O2v2 | O2afg | Very high | O2v2 |  |  |
| KPN0019; MGH sepsis isolate | *Klebsiella pneumoniae* | ST2440 | KL116 | unknown (KL116) | O1/O2v1 | O2a | Very high | O2v1 |  |  |
| KPN0020; MGH sepsis isolate | *Klebsiella pneumoniae* | ST35 | KL22 | K22 | O1/O2v1 | O1 | Very high | O1v1 |  |  |
| KPN0021; MGH sepsis isolate | *Klebsiella pneumoniae* | ST1838 | KL14 | K14 | O3b | O3b | Very high | O3b |  |  |
| KPN0022; MGH sepsis isolate | *Klebsiella pneumoniae* | ST37 | KL38 | K38 | O3b | O3b | Very high | O3b |  |  |
| KPN0023; MGH sepsis isolate | *Klebsiella pneumoniae* | ST3417-1LV | KL169 | unknown (KL169) | OL104 | unknown (OL104) | Good | Non-O1, O2, O3, O5 |  |  |
| KPN0024; MGH sepsis isolate | *Klebsiella pneumoniae* | ST3994 | KL2 | K2 | O1/O2v1 | O1 | Very high | O1v1 |  |  |
| KPN0025; MGH sepsis isolate | *Klebsiella pneumoniae* | ST20 | KL28 | K28 | O1/O2v2 | O1 | Very high | O1v2 |  |  |
| KPN0026; MGH sepsis isolate | *Klebsiella pneumoniae* | ST883 | unknown (KL107) | unknown (KL107) | unknown (O4) | unknown (O4) | None | Non-O1, O2, O3, O5 |  |  |
| KPN0027; MGH sepsis isolate | *Klebsiella pneumoniae* | ST323 | KL21 | K21 | O3b | O3b | Very high | O3b |  |  |
| KPN0028; MGH sepsis isolate | *Klebsiella pneumoniae* | ST528 | KL128 | unknown (KL128) | O3b | O3b | Very high | O3b |  |  |
| KPN0029; MGH sepsis isolate | *Klebsiella pneumoniae* | ST86 | KL2 | K2 | O1/O2v1 | O1 | Very high | O1v1 |  |  |
| KPN0030; MGH sepsis isolate | *Klebsiella pneumoniae* | ST17-1LV | KL122 | unknown (KL122) | O1/O2v2 | O2afg | Very high | O2v2 |  |  |
| KPN0031; MGH sepsis isolate | *Klebsiella pneumoniae* | ST14 | KL2 | K2 | O1/O2v1 | O1* | Very high | O1v1 |  |  |
| KPN0032; MGH sepsis isolate | *Klebsiella pneumoniae* | ST1076 | KL137 | unknown (KL137) | OL101 | unknown (OL101) | Good | Non-O1, O2, O3, O5 |  |  |
| KPN0033; MGH sepsis isolate | *Klebsiella pneumoniae* | ST45 | KL127 | unknown (KL127) | OL101 | unknown (OL101) | Good | Non-O1, O2, O3, O5 |  |  |
| KPN0034; MGH sepsis isolate | *Klebsiella pneumoniae* | ST29-1LV | KL54 | K54 | O1/O2v2 | O1 | Very high | O1v2 |  |  |
| KPN0035; MGH sepsis isolate | *Klebsiella pneumoniae* | ST86 | KL2 | K2 | O1/O2v1 | O1 | Very high | O1v1 |  |  |
| KPN0036; MGH sepsis isolate | *Klebsiella pneumoniae* | ST987 | KL46 | K46 | O3b | O3b | Good | O3b |  |  |
| KPN0037; MGH sepsis isolate | *Klebsiella pneumoniae* | ST147 | KL64 | K64 | O1/O2v1 | O2a | Good | O2v1 |  |  |
| KPN0038; MGH sepsis isolate | *Klebsiella pneumoniae* | ST1213 | KL103 | unknown (KL103) | O1/O2v1 | O1 | Very high | O1v1 |  |  |
| KPN0039; MGH sepsis isolate | *Klebsiella pneumoniae* | ST483 | KL110 | unknown (KL110) | O3b | O3b | Good | O3b |  |  |
| KPN0040; MGH sepsis isolate | *Klebsiella pneumoniae* | ST111 | KL113 | unknown (KL113) | O1/O2v2 | O1 | Very high | O1v2 |  |  |
| KPN0041; MGH sepsis isolate | *Klebsiella pneumoniae* | ST3430 | KL52 | K52 | OL101 | unknown (OL101) | Good | Non-O1, O2, O3, O5 |  |  |
| KPN0042; MGH sepsis isolate | *Klebsiella pneumoniae* | ST14 | KL2 | K2 | O1/O2v1 | O1 | Very high | O1v1 |  |  |
| KPN0043; MGH sepsis isolate | *Klebsiella pneumoniae* | ST39 | KL62 | K62 | O1/O2v2 | O1 | Very high | O1v2 |  |  |
| KPN0044; MGH sepsis isolate | *Klebsiella pneumoniae* | ST277 | unknown (KL46) | unknown (K46) | O3b | O3b | Good | O3b |  |  |
| KPN0045; MGH sepsis isolate | *Klebsiella pneumoniae* | ST34 | KL174 | unknown (KL174) | O1/O2v2 | O1 | Very high | O1v2 |  |  |
| KPN0046; MGH sepsis isolate | *Klebsiella pneumoniae* | ST483 | KL110 | unknown (KL110) | O3b | O3b | Good | O3b |  |  |
| KPN0047; MGH sepsis isolate | *Klebsiella variicola subsp. variicola* | ST4169 | KL143 | unknown (KL143) | O3/O3a | O3/O3a | Very high | O3a |  |  |
| KPN0048; MGH sepsis isolate | *Klebsiella quasipneumoniae subsp. similipneumoniae* | ST5715-1LV | KL139 | unknown (KL139) | O4 | O4 | Very high | Non-O1, O2, O3, O5 |  |  |
| KPN0049; MGH sepsis isolate | *Klebsiella quasipneumoniae subsp. quasipneumoniae* | ST668 | KL142 | unknown (KL142) | O5 | O5 | High | O5 |  |  |
| KPN0050; MGH sepsis isolate | *Klebsiella pneumoniae* | ST831 | KL18 | K18 | O1/O2v1 | O1 | Very high | O1v1 |  |  |
| KPN0051; MGH sepsis isolate | *Klebsiella variicola subsp. variicola* | ST595 | KL16 | K16 | O5 | O5 | Very high | O5 |  |  |
| KPN0052; MGH sepsis isolate | *Klebsiella pneumoniae* | ST323 | KL21 | K21 | O3b | O3b | Very high | O3b |  |  |
| KPN0053; MGH sepsis isolate | *Klebsiella pneumoniae* | ST1621-1LV | KL46 | K46 | O3b | O3b | Very high | O3b |  |  |
| KPN0054; MGH sepsis isolate | *Klebsiella pneumoniae* | ST45 | KL24 | K24 | O1/O2v1 | O2a | Very high | O2v1 |  |  |
| KPN0055; MGH sepsis isolate | *Klebsiella pneumoniae* | ST3660 | KL25 | K25 | O1/O2v1 | O1 | Very high | O1v1 |  |  |
| KPN0057; MGH sepsis isolate | *Klebsiella pneumoniae* | ST337 | KL174 | unknown (KL174) | O1/O2v2 | O1 | Very high | O1v2 |  |  |
| KPN0058; MGH sepsis isolate | *Klebsiella pneumoniae* | ST307 | KL102 | unknown (KL102) | O1/O2v2 | O2afg | Very high | O2v2 |  |  |
| KPN0059; MGH sepsis isolate | *Klebsiella pneumoniae* | ST395 | KL108 | unknown (KL108) | O1/O2v2 | O1 | Very high | O1v2 |  |  |
| KPN0060; MGH sepsis isolate | *Klebsiella pneumoniae* | ST36 | KL27 | K27 | O1/O2v2 | O2afg | Very high | O2v2 |  |  |
| KPN0061; MGH sepsis isolate | *Klebsiella pneumoniae* | ST134 | KL25 | K25 | O5 | O5 | High | O5 |  |  |
| KPN0062; MGH sepsis isolate | *Klebsiella quasipneumoniae subsp. similipneumoniae* | ST582 | KL121 | unknown (KL121) | O5 | O5 | Good | O5 |  |  |
| KPN0063; MGH sepsis isolate | *Klebsiella pneumoniae* | ST661 | KL39 | K39 | O3/O3a | O3/O3a | Very high | O3a |  |  |
| KPN0064; MGH sepsis isolate | *Klebsiella pneumoniae* | ST857 | KL13 | K13 | O3b | O3b | Very high | O3b |  |  |
| KPN0065; MGH sepsis isolate | *Klebsiella pneumoniae* | ST20 | KL28 | K28 | O1/O2v2 | O1 | Very high | O1v2 |  |  |
| KPN0066; MGH sepsis isolate | *Klebsiella pneumoniae* | ST307 | KL102 | unknown (KL102) | O1/O2v2 | O2afg | Very high | O2v2 |  |  |
| KPN0067; MGH sepsis isolate | *Klebsiella pneumoniae* | ST36 | KL61 | K61 | O1/O2v1 | O2a | Good | O2v1 |  |  |
| KPN0069; MGH sepsis isolate | *Klebsiella pneumoniae* | ST111 | KL63 | K63 | O1/O2v2 | O1 | Very high | O1v2 |  |  |
| KPN0070; MGH sepsis isolate | *Klebsiella pneumoniae* | ST327 | KL39 | K39 | O1/O2v1 | O1 | Very high | O1v1 |  |  |
| KPN0071; MGH sepsis isolate | *Klebsiella pneumoniae* | ST111 | KL63 | K63 | O1/O2v2 | O1 | Very high | O1v2 |  |  |
| KPN0072; MGH sepsis isolate | *Klebsiella pneumoniae* | ST134 | KL25 | K25 | O5 | O5 | High | O5 |  |  |
| KPN0073; MGH sepsis isolate | *Klebsiella pneumoniae* | ST1 | KL45 | K45 | O1/O2v2 | O1 | Very high | O1v2 |  |  |
| KPN0074; MGH sepsis isolate | *Klebsiella pneumoniae* | ST1661-1LV | KL117 | unknown (KL117) | O1/O2v2 | O1 | Very high | O1v2 |  |  |
| KPN0075; MGH sepsis isolate | *Klebsiella pneumoniae* | ST54 | KL14 | K14 | O3b | O3b | High | O3b |  |  |
| KPN0076; MGH sepsis isolate | *Klebsiella pneumoniae* | ST17 | KL25 | K25 | O5 | O5 | Good | O5 |  |  |
| KPN0077; MGH sepsis isolate | *Klebsiella pneumoniae* | ST29 | KL54 | K54 | O1/O2v2 | O1 | Very high | O1v2 |  |  |
| KPN0078; MGH sepsis isolate | *Klebsiella variicola subsp. variicola* | ST2631-1LV | KL134 | unknown (KL134) | O3/O3a | O3/O3a | Very high | O3a |  |  |
| KPN0079; MGH sepsis isolate | *Klebsiella pneumoniae* | ST12 | KL122 | unknown (KL122) | O1/O2v2 | O2afg | Good | O2v2 |  |  |
| KPN0080; MGH sepsis isolate | *Klebsiella pneumoniae* | ST307 | KL102 | unknown (KL102) | O1/O2v2 | O2afg | Very high | O2v2 |  |  |
| KPN0081; MGH sepsis isolate | *Klebsiella pneumoniae* | ST4283 | KL145 | unknown (KL145) | O4 | O4 | Very high | Non-O1, O2, O3, O5 |  |  |
| KPN0082; MGH sepsis isolate | *Klebsiella quasipneumoniae subsp. similipneumoniae* | ST582 | KL121 | unknown (KL121) | O5 | O5 | Good | O5 |  |  |
| KPN0083; MGH sepsis isolate | *Klebsiella variicola subsp. variicola* | ST4750-1LV | KL3 | K3 | O3/O3a | O3/O3a | Good | O3a |  |  |
| MGH: Massachusetts General Hospital |  |  |  |  |  |  |  |  |  |  |
| * The original Kaptive call for KPN0031 of O2a was corrected to O1 after determining that the wbbY gene was present but split across two contigs and each segment length was under the detection threshold for Kaptive. | | | | | | |  |  |  |  |

**Table S2**. Sequence Accession IDs used in the design

**O1/2, wzm:** CP067927.1, CP067622.1, CP031798.1, CP096260.1, CP031808.1, CP052386.1, CP054268.1, CP052524.1, LR890735.1, CP078770.1, CP054780.1, CP052036.1, CP073783.1, CP052268.1, CP062138.1, CP078773.1, CP052716.1, CP043669.1

**O1, wbbY:** MG458672.1

**O2afg, gmlC:** CP091822.1, CP009863.1, CP021955.1, CP025092.1, CP009114.1, CP065343.1, CP015822.1, CP020841.1, CP030070.1, CP032175.1, CP035531.1, CP036320.1, CP052706.1, CP002910.1, CP022823.1, CP052150.1, CP078754.1, CP018337.1, CP052172.1, CP087122.1, CP037441.1, LR890281.1

**O3, wzm:** AB795941.1, LT174603.1, LR890598.1, LR890512.1, LR890215.1, AB010296.1, CP050829.1, CP053364.1, CP086285.1, CP063934.1, LT795501.1, LT795500.1, CP041644.1, CP052324.1, CP034359.1, CP039828.1, CP082799.1, CP041934.1, CP041092.1, LR134206.1, CP024515.1, CP006656.1, CP094237.1, CP067882.1, CP085478.1

**O3b, wbdD:** CP052324.1, CP041934.1, LR596814.1, CP031938.1, CP018306.1, CP058960.1, CP090519.1, CP052265.1, CP039828.1, CP070586.1, CP031577.1, LR133932.1, CP057330.1, LS483483.1, CP033844.1, LT174599.1, CP018816.1, CP090245.1, CP070020.1, LR134333.1

**O5, wzt:** CP075885.1, CP068237.1, LR130543.1, CP028555.2, LR890698.1, AF189151.1, CP043932.1

**
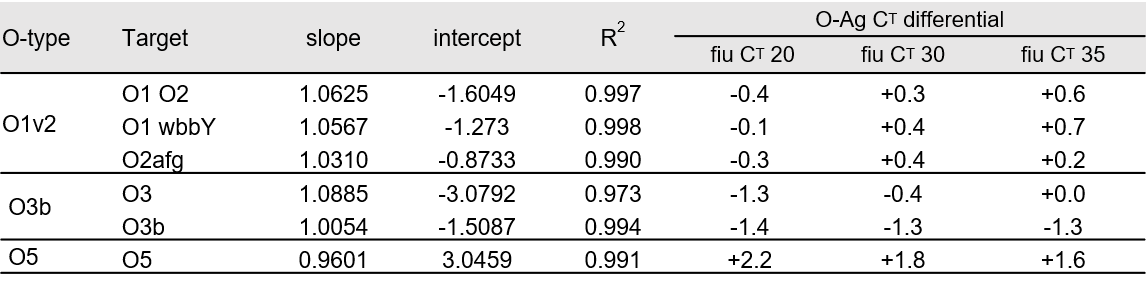
**

C)

A)

B)

D)

**Figure S1. Correlation of Kp qPCR set with O-typing real-time PCR sets.**

Linearity of C*_T_* values for Kp fiu set with O-typing real-time PCR for O1v2-type (A), O3b-type (B) and O5-type (C) strains. Linear regression data and estimated C*_T_* value differential for O-antigens based on Kp *fiu* C*_T_* values of 20, 30 and 35.


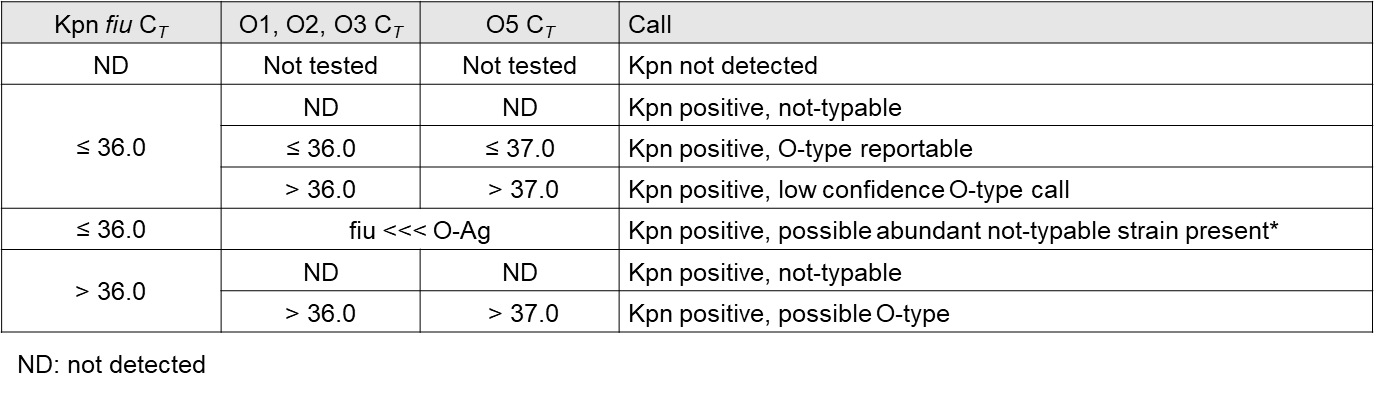


**Table S3.** Call reporting scheme for direct real-time PCR testing of stool samples.

**Table S4.** Method comparison data for direct testing of stool samples.

|  | **Kp qPCR** | | | | **O-Ag real-time PCR** | | | | | | | | | | | **Culture + Endpoint O-typing PCR** | | | | | | | | **Discrepant Investig.** | |
| --- | --- | --- | --- | --- | --- | --- | --- | --- | --- | --- | --- | --- | --- | --- | --- | --- | --- | --- | --- | --- | --- | --- | --- | --- | --- |
| **SID** | **23S C*_T_*** | **Kpn**  **fiu**  **C*_T_*** | **Kp** | **% Kp** | **O1/O2 C*_T_*** | **O1 wbbY**  **C*_T_*** | **O2afg**  **C*_T_*** | **O3 C*_T_*** | **O3b**  **C*_T_*** | **O5 C*_T_*** | **1° Call** | **2° Call** | **3° Call** | **fiu - O-Ag ΔC***_T_* | **Note** | **Kp** | **O1/2** | **O1**  **wbbY** | **O3** | **O5** | **1° Call** | **2° Call** | **3° Call** | **O4, O8, O9, O12** | **plate streak PCR** |
| MGHK  001 | 12.2 | 34.3 | D | 2.5E-04 | 34.3 | 32.3 | 32.3 | ND | ND | ND | O1v2 | ND | ND | < 5 | None | ND | NT | NT | NT | NT | NT | NT | NT | NT | NT |
| MGHK  002 | 11.9 | 33.8 | D | 2.8E-04 | ND | ND | ND | 36.0 | 32.8 | ND | O3b | ND | ND | < 5 | None | D | NEG | NT | POS | NEG | O3 | ND | ND | NT | NT |
| MGHK  003 | 17.3 | 22.9 | D | 2.3E+01 | ND | ND | ND | 30.1 | ND | ND | O3a | ND | ND | 7.2 | possible non-typable Kp present | D | NEG | NT | POS | NEG | O3 | ND | ND | NEG | NT |
| MGHK  004 | 11.3 | 30.9 | D | 1.4E-03 | ND | ND | ND | 30.5 | ND | 36.4 | O3a | O5 | ND | < 5 | None | D | NEG | NT | POS | POS | O3 | O5 | ND | NT | NT |
| MGHK  005 | 17.7 | ND | ND | ND | NT | NT | NT | NT | NT | NT | NT | NT | NT | NA | None | ND | NT | NT | NT | NT | NT | NT | NT | NT | NT |
| MGHK 006 | 11.5 | 34.8 | D | 1.1E-04 | ND | ND | ND | 33.3 | 32.3 | ND | O3b | ND | ND | < 5 | None | D | NEG | NT | POS | POS | O3 | O5 | ND | NT | NT |
| MGHK 007 | 21.4 | ND | ND | ND | NT | NT | NT | NT | NT | NT | NT | NT | NT | NA | None | ND | NT | NT | NT | NT | NT | NT | NT | NT | NT |
| MGHK 008 | 12.8 | 34.1 | D | 4.6E-04 | 33.2 | 31.9 | 33.8 | ND | ND | ND | O1v2 | ND | ND | < 5 | None | D | POS | POS | NEG | NEG | O1 | ND | ND | NT | NT |
| MGHK 009 | 17.4 | ND | ND | ND | NT | NT | NT | NT | NT | NT | NT | NT | NT | NA | None | ND | NT | NT | NT | NT | NT | NT | NT | NT | NT |
| MGHK 010 | 15.7 | ND | ND | ND | NT | NT | NT | NT | NT | NT | NT | NT | NT | NA | None | ND | NT | NT | NT | NT | NT | NT | NT | NT | NT |
| MGHK 011 | 13.1 | ND | ND | ND | NT | NT | NT | NT | NT | NT | NT | NT | NT | NA | None | ND | NT | NT | NT | NT | NT | NT | NT | NT | NT |
| MGHK 012 | 11.5 | 34.0 | D | 1.9E-04 | ND | ND | ND | 34.9 | 30.8 | ND | O3b | ND | ND | < 5 | None | D | NEG | NT | POS | NEG | O3 | ND | ND | NT | NT |
| MGHK 013 | 10.2 | 16.8 | D | 1.1E+01 | 21.0 | 20.6 | 20.6 | 32.6 | 35.7 | 19.8 | O1v2 | O5 | O3b | < 5 | None | D | POS | POS | NEG | POS | O1 | O5 | ND | NT | O1 + O3 + O5 |
| MGHK 014 | 13.2 | ND | ND | ND | NT | NT | NT | NT | NT | NT | NT | NT | NT | NA | None | ND | NT | NT | NT | NT | NT | NT | NT | NT | NT |
| MGHK 015 | 12.6 | 18.7 | D | 1.6E+01 | 30.0 | ND | 26.4 | 20.6 | 16.8 | ND | O3b | O2v2 | ND | < 5 | None | D | NEG | NT | POS | NEG | O3 | ND | ND | NT | NT |
| MGHK 016 | 10.9 | 38.7 | > CO | 4.9E-06 | NT | NT | NT | NT | NT | NT | NT | NT | NT | NA | None | D | NEG | NT | POS | NEG | O3 | ND | ND | NT | NT |
| MGHK 017 | 13.8 | 30.8 | D | 8.7E-03 | 29.5 | 29.0 | ND | ND | ND | ND | O1v1 | ND | ND | < 5 | None | D | POS | POS | NEG | NEG | O1 | ND | ND | NT | NT |
| MGHK 018 | 14.7 | ND | ND | ND | NT | NT | NT | NT | NT | NT | NT | NT | NT | NA | None | ND | NT | NT | NT | NT | NT | NT | NT | NT | NT |
| MGHK 019 | 13.9 | 22.9 | D | 2.4E+00 | ND | ND | ND | ND | ND | 25.5 | O5 | ND | ND | < 5 | None | D | NEG | NT | NEG | POS | O5 | ND | ND | NT | NT |
| MGHK 020 | 23.2 | ND | ND | ND | NT | NT | NT | NT | NT | NT | NT | NT | NT | NA | None | ND | NT | NT | NT | NT | NT | NT | NT | NT | NT |
| MGHK 021 | 12.7 | ND | ND | ND | NT | NT | NT | NT | NT | NT | NT | NT | NT | NA | None | ND | NT | NT | NT | NT | NT | NT | NT | NT | NT |
| MGHK 022 | 18.6 | ND | ND | ND | NT | NT | NT | NT | NT | NT | NT | NT | NT | NA | None | ND | NT | NT | NT | NT | NT | NT | NT | NT | NT |
| MGHK 023 | 11.4 | 33.2 | D | 3.2E-04 | ND | ND | ND | ND | ND | 35.1 | O5 | ND | ND | < 5 | None | D | NEG | NT | NEG | POS | O5 | ND | ND | NT | NT |
| MGHK 024 | 23.9 | ND | ND | ND | NT | NT | NT | NT | NT | NT | NT | NT | NT | NA | None | ND | NT | NT | NT | NT | NT | NT | NT | NT | NT |
| MGHK 025 | 11.7 | 21.7 | D | 1.1E+00 | 21.4 | ND | 35.5 | ND | ND | ND | O2v1 | ND | ND | < 5 | None | D | POS | NEG | NEG | NEG | O2 | ND | ND | NT | NT |
| MGHK 026 | 17.2 | 24.4 | D | 7.3E+00 | 23.4 | ND | 22.7 | ND | ND | ND | O2v2 | ND | ND | < 5 | None | D | POS | NEG | NEG | NEG | O2 | ND | ND | NT | NT |
| MGHK 027 | 13.5 | 22.8 | D | 1.8E+00 | 22.2 | 22.5 | 22.4 | 34.2 | 29.8 | 26.9 | O1v2 | O5 | O3b | < 5 | None | D | POS | POS | NEG | POS | O1 | O5 | ND | NT | O1 + O3 + O5 |
| MGHK 028 | 17.1 | ND | ND | ND | NT | NT | NT | NT | NT | NT | NT | NT | NT | NA | None | ND | NT | NT | NT | NT | NT | NT | NT | NT | NT |
| MGHK 029 | 11.4 | ND | ND | ND | NT | NT | NT | NT | NT | NT | NT | NT | NT | NA | None | ND | NT | NT | NT | NT | NT | NT | NT | NT | NT |
| MGHK 030 | 12.3 | 26.7 | D | 5.3E-02 | ND | ND | ND | 36.7 | 31.5 | ND | O3b | ND | ND | < 5 | None | D | NEG | NT | POS | NEG | O3 | ND | ND | NT | NT |
| MGHK 031 | 15.2 | ND | ND | ND | NT | NT | NT | NT | NT | NT | NT | NT | NT | NA | None | ND | NT | NT | NT | NT | NT | NT | NT | NT | NT |
| MGHK 032 | 11.1 | ND | ND | ND | NT | NT | NT | NT | NT | NT | NT | NT | NT | NA | None | ND | NT | NT | NT | NT | NT | NT | NT | NT | NT |
| MGHK 033 | 13.1 | 26.9 | D | 7.8E-02 | 25.6 | 25.1 | ND | ND | ND | ND | O1v1 | ND | ND | < 5 | None | D | POS | POS | NEG | NEG | O1 | ND | ND | NT | NT |
| MGHK 034 | 11.2 | ND | ND | ND | NT | NT | NT | NT | NT | NT | NT | NT | NT | NA | None | ND | NT | NT | NT | NT | NT | NT | NT | NT | NT |
| MGHK 035 | 20.0 | 35.4 | D | 2.5E-02 | ND | ND | ND | ND | ND | ND | Not typed | ND | ND | ND | None | ND | NT | NT | NT | NT | NT | NT | NT | NT | NT |
| MGHK 036 | 13.8 | ND | ND | ND | NT | NT | NT | NT | NT | NT | NT | NT | NT | NA | None | ND | NT | NT | NT | NT | NT | NT | NT | NT | NT |
| MGHK 037 | 14.2 | 35.3 | D | 5.0E-04 | ND | ND | ND | ND | ND | ND | Not typed | ND | ND | ND | None | D | NEG | NT | NEG | NEG | not typed | ND | ND | NEG | NT |
| MGHK 038 | 13.5 | 25.0 | D | 4.0E-01 | 22.8 | ND | 22.1 | ND | ND | ND | O2v2 | ND | ND | < 5 | None | D | POS | NEG | NEG | NEG | O2 | ND | ND | NT | NT |
| MGHK 039 | 24.2 | ND | ND | ND | NT | NT | NT | NT | NT | NT | NT | NT | NT | NA | None | ND | NT | NT | NT | NT | NT | NT | NT | NT | NT |
| MGHK 040 | 11.4 | 30.1 | D | 2.7E-03 | 28.9 | 28.5 | ND | 39.4 | 34.5 | ND | O1v1 | O3b | None | < 5 | None | ND | NT | NT | NT | NT | NT | NT | NT | NT | NT |
| MGHK 041 | 14.1 | 37.6 | > CO | 9.4E-05 | NT | NT | NT | NT | NT | NT | NT | NT | NT | NA | None | ND | NT | NT | NT | NT | NT | NT | NT | NT | NT |
| MGHK 042 | 18.1 | 35.3 | D | 7.2E-03 | ND | ND | ND | 37.8 | 32.3 | ND | O3b | ND | ND | < 5 | None | D | POS | POS | POS | NEG | O1 | O3 | ND | NT | NT |
| MGHK 043 | 14.4 | ND | ND | ND | NT | NT | NT | NT | NT | NT | NT | NT | NT | NA | None | ND | NT | NT | NT | NT | NT | NT | NT | NT | NT |
| MGHK 044 | 15.2 | ND | ND | ND | NT | NT | NT | NT | NT | NT | NT | NT | NT | NA | None | ND | NT | NT | NT | NT | NT | NT | NT | NT | NT |
| MGHK 045 | 11.3 | 37.8 | > CO | 1.2E-05 | NT | NT | NT | NT | NT | NT | NT | NT | NT | NA | None | D | POS | POS | NEG | NEG | O1 | ND | ND | NT | not typable |
| MGHK 046 | 13.0 | ND | ND | ND | NT | NT | NT | NT | NT | NT | NT | NT | NT | NA | None | ND | NT | NT | NT | NT | NT | NT | NT | NT | NT |
| MGHK 047 | 11.8 | 38.4 | > CO | 1.1E-05 | NT | NT | NT | NT | NT | NT | NT | NT | NT | NA | None | ND | NT | NT | NT | NT | NT | NT | NT | NT | NT |
| MGHK 048 | 13.4 | ND | ND | ND | NT | NT | NT | NT | NT | NT | NT | NT | NT | NA | None | ND | NT | NT | NT | NT | NT | NT | NT | NT | NT |
| MGHK 049 | 12.8 | ND | ND | ND | NT | NT | NT | NT | NT | NT | NT | NT | NT | NA | None | ND | NT | NT | NT | NT | NT | NT | NT | NT | NT |
| MGHK 050 | 19.6 | 32.8 | D | 1.2E-01 | 32.6 | ND | 32.4 | ND | ND | ND | O2v2 | ND | ND | < 5 | None | ND | NT | NT | NT | NT | NT | NT | NT | NT | NT |
| MGHK 051 | 12.7 | 24.0 | D | 4.7E-01 | ND | 19.0 | ND | 23.3 | ND | ND | O3a | ND | ND | < 5 | None | D | NEG | NT | POS | NEG | O3 | ND | ND | NT | NT |
| MGHK 052 | 12.7 | ND | ND | ND | NT | NT | NT | NT | NT | NT | NT | NT | NT | NA | None | ND | NT | NT | NT | NT | NT | NT | NT | NT | NT |
| MGHK 053 | 17.8 | ND | ND | ND | NT | NT | NT | NT | NT | NT | NT | NT | NT | NA | None | ND | NT | NT | NT | NT | NT | NT | NT | NT | NT |
| MGHK 054 | 17.1 | 28.0 | D | 6.3E-01 | ND | ND | ND | ND | ND | 29.8 | O5 | ND | ND | < 5 | None | D | NEG | NT | NEG | POS | O5 | ND | ND | NT | NT |
| MGHK 055 | 13.3 | ND | ND | ND | NT | NT | NT | NT | NT | NT | NT | NT | NT | NA | None | ND | NT | NT | NT | NT | NT | NT | NT | NT | NT |
| MGHK 056 | 11.8 | ND | ND | ND | NT | NT | NT | NT | NT | NT | NT | NT | NT | NA | None | ND | NT | NT | NT | NT | NT | NT | NT | NT | NT |
| MGHK 057 | 12.6 | 19.4 | D | 1.0E+01 | 18.1 | ND | 17.8 | 21.0 | 19.2 | ND | O2v2 | O3b | ND | < 5 | None | D | POS | NEG | POS | NEG | O2 | O3 | ND | NT | NT |
| MGHK 058 | 14.0 | ND | ND | ND | NT | NT | NT | NT | NT | NT | NT | NT | NT | NA | None | ND | NT | NT | NT | NT | NT | NT | NT | NT | NT |
| MGHK 059 | 16.7 | ND | ND | ND | NT | NT | NT | NT | NT | NT | NT | NT | NT | NA | None | ND | NT | NT | NT | NT | NT | NT | NT | NT | NT |
| MGHK 060 | 10.7 | 25.9 | D | 3.0E-02 | 20.5 | ND | 19.6 | 23.8 | 21.2 | ND | O2v2 | O3b | ND | < 5 | None | D | POS | NEG | NEG | NEG | O2 | ND | ND | NT | O2 + O3 |
| MGHK 061 | 13.2 | ND | ND | ND | NT | NT | NT | NT | NT | NT | NT | NT | NT | NA | None | ND | NT | NT | NT | NT | NT | NT | NT | NT | NT |
| MGHK 062 | 11.9 | 34.1 | D | 2.3E-04 | 31.3 | ND | ND | ND | ND | ND | O2v1 | ND | ND | < 5 | None | D | POS | NEG | NEG | NEG | O2 | ND | ND | NT | NT |
| MGHK 063 | 14.5 | ND | ND | ND | NT | NT | NT | NT | NT | NT | NT | NT | NT | NA | None | ND | NT | NT | NT | NT | NT | NT | NT | NT | NT |
| MGHK 064 | 12.0 | 20.7 | D | 2.7E+00 | 18.9 | 18.9 | 24.1 | 31.8 | ND | ND | O1v2 | O3a | ND | < 5 | None | D | POS | POS | NEG | NEG | O1 | ND | ND | NT | O1 |
| MGHK 065 | 14.7 | 23.6 | D | 2.4E+00 | 22.1 | 21.8 | 21.4 | ND | ND | ND | O1v2 | ND | ND | < 5 | None | ND | NT | NT | NT | NT | NT | NT | NT | NT | NT |
| MGHK 066 | 20.0 | ND | ND | ND | NT | NT | NT | NT | NT | NT | NT | NT | NT | NA | None | ND | NT | NT | NT | NT | NT | NT | NT | NT | NT |
| MGHK 067 | 13.1 | 38.4 | > CO | 2.8E-05 | NT | NT | NT | NT | NT | NT | NT | NT | NT | NA | None | ND | NT | NT | NT | NT | NT | NT | NT | NT | NT |
| MGHK 068 | 28.0 | ND | ND | ND | NT | NT | NT | NT | NT | NT | NT | NT | NT | NA | None | ND | NT | NT | NT | NT | NT | NT | NT | NT | NT |
| MGHK 069 | 16.1 | 35.2 | D | 1.9E-03 | 37.0 | 35.2 | ND | ND | ND | ND | O1v2 | ND | ND | < 5 | None | D | POS | POS | NEG | NEG | O1 | ND | ND | NT | NT |
| MGHK 070 | 10.9 | 26.0 | D | 3.3E-02 | 28.2 | ND | 27.2 | 26.2 | ND | ND | O2v2 | O3a | ND | < 5 | None | D | POS | NEG | POS | NEG | O2 | O3 | ND | NT | NT |
| MGHK 071 | 12.5 | ND | ND | ND | NT | NT | NT | NT | NT | NT | NT | NT | NT | NA | None | ND | NT | NT | NT | NT | NT | NT | NT | NT | NT |
| MGHK 072 | 14.4 | 26.9 | D | 2.0E-01 | 24.2 | 31.2 | 24.5 | 27.0 | 30.9 | ND | O2v2 | O3b | ND | < 5 | None | D | POS | NEG | POS | NEG | O2 | O3 | ND | NT | NT |
| MGHK 073 | 16.0 | 36.7 | > CO | 6.9E-04 | NT | NT | NT | NT | NT | NT | NT | NT | NT | NA | None | D | POS | POS | NEG | NEG | O1 | ND | ND | NT | NT |
| MGHK 074 | 12.6 | ND | ND | ND | NT | NT | NT | NT | NT | NT | NT | NT | NT | NA | None | ND | NT | NT | NT | NT | NT | NT | NT | NT | NT |
| MGHK 075 | 13.9 | ND | ND | ND | NT | NT | NT | NT | NT | NT | NT | NT | NT | NA | None | ND | NT | NT | NT | NT | NT | NT | NT | NT | NT |
| MGHK 076 | 11.6 | ND | ND | ND | NT | NT | NT | NT | NT | NT | NT | NT | NT | NA | None | ND | NT | NT | NT | NT | NT | NT | NT | NT | NT |
| MGHK 077 | 12.6 | 22.8 | D | 1.0E+00 | 28.1 | ND | 26.8 | ND | ND | 33.4 | O2v2 | O5 | ND | 5.3 | possible non-typable Kp present | D | NEG | NT | NEG | NEG | not typed | ND | ND | O9, O12 | NT |
| MGHK 078 | 9.2 | 19.3 | D | 1.1E+00 | ND | ND | ND | ND | ND | ND | Not typed | ND | ND | ND | None | D | NEG | NT | POS | NEG | O3 | ND | ND | O9 | NT |
| MGHK 079 | 12.9 | 24.1 | D | 5.0E-01 | ND | ND | ND | ND | ND | 30.6 | O5 | ND | ND | 6.5 | possible non-typable Kp present | D | NEG | NT | NEG | POS | O5 | ND | O5 | NT | NT |
| MGHK 080 | 12.3 | 24.0 | D | 3.4E-01 | 24.4 | 23.8 | ND | 26.6 | 31.6 | 28.8 | O1v1 | O3b | O5 | < 5 | None | D | POS | POS | POS | POS | O1 | O3 | O5 | NT | NT |
| MGHK 081 | 11.0 | 19.6 | D | 2.9E+00 | 22.8 | 22.0 | 21.8 | 18.4 | 17.1 | ND | O1v2 | O3b | ND | < 5 | None | D | POS | POS | POS | NEG | O1 | O3 | ND | NT | NT |
| MGHK 082 | 15.3 | 37.8 | > CO | 2.0E-04 | NT | NT | NT | NT | NT | NT | NT | NT | NT | NA | None | ND | NT | NT | NT | NT | NT | NT | NT | NT | NT |
| MGHK 083 | 12.0 | 32.2 | D | 9.0E-04 | ND | ND | ND | ND | ND | ND | Not typed | ND | ND | ND | None | ND | NT | NT | NT | NT | NT | NT | NT | NT | NT |
| MGHK 084 | 12.0 | ND | ND | ND | NT | NT | NT | NT | NT | NT | NT | NT | NT | NA | None | ND | NT | NT | NT | NT | NT | NT | NT | NT | NT |
| MGHK 085 | 15.8 | ND | ND | ND | NT | NT | NT | NT | NT | NT | NT | NT | NT | NA | None | ND | NT | NT | NT | NT | NT | NT | NT | NT | NT |
| MGHK 086 | 16.0 | 34.1 | D | 4.2E-03 | ND | ND | ND | ND | ND | ND | Not typed | ND | ND | ND | None | ND | NT | NT | NT | NT | NT | NT | NT | NT | NT |
| MGHK 087 | 14.7 | ND | ND | ND | NT | NT | NT | NT | NT | NT | NT | NT | NT | NA | None | ND | NT | NT | NT | NT | NT | NT | NT | NT | NT |
| MGHK 088 | 11.0 | 17.1 | D | 1.7E+01 | 14.8 | 15.4 | 16.8 | 21.0 | 19.9 | 26.6 | O1v2 | O3b | O5 | < 5 | None | D | POS | POS | NEG | NEG | O1 | ND | ND | NT | O1 + O3 |
| MGHK 089 | 15.1 | 29.2 | D | 6.7E-02 | ND | ND | ND | 29.9 | ND | ND | O3a | ND | ND | < 5 | None | ND | NT | NT | NT | NT | NT | NT | NT | NT | NT |
| MGHK 090 | 11.9 | 36.9 | > CO | 3.3E-05 | NT | NT | NT | NT | NT | NT | NT | NT | NT | NA | None | D | POS | POS | POS | NEG | O1 | O3 | ND | NT | NT |
| MGHK 091 | 15.4 | 38.0 | > CO | 1.8E-04 | NT | NT | NT | NT | NT | NT | NT | NT | NT | NA | None | ND | NT | NT | NT | NT | NT | NT | NT | NT | NT |
| MGHK 092 | 11.5 | 19.5 | D | 4.5E+00 | 35.2 | 35.2 | ND | 31.1 | 28.3 | 34.3 | O3b | O1v1 | O5 | 8.9 | possible non-typable Kp present | D | NEG | NT | POS | NEG | O3 | ND | ND | NEG | O3 |
| MGHK 093 | 11.4 | 36.9 | > CO | 2.4E-05 | NT | NT | NT | NT | NT | NT | NT | NT | NT | NA | None | D | NEG | NT | POS | NEG | O3 | ND | ND | NT | NT |
| MGHK 094 | 12.6 | 18.9 | D | 1.5E+01 | ND | ND | ND | 18.3 | ND | ND | O3a | ND | ND | < 5 | None | D | NEG | NT | POS | NEG | O3 | ND | ND | NT | NT |
| MGHK 095 | 16.1 | ND | ND | ND | NT | NT | NT | NT | NT | NT | NT | NT | NT | NA | None | ND | NT | NT | NT | NT | NT | NT | NT | NT | NT |
| MGHK 096 | 18.3 | 24.2 | D | 2.9E+00 | 22.7 | 21.9 | ND | ND | ND | ND | O1v1 | ND | ND | < 5 | None | D | POS | POS | POS | NEG | O1 | O3 | ND | NT | NT |
| MGHK 097 | 11.9 | ND | ND | ND | NT | NT | NT | NT | NT | NT | NT | NT | NT | NA | None | ND | NT | NT | NT | NT | NT | NT | NT | NT | NT |
| MGHK 098 | 11.6 | ND | ND | ND | NT | NT | NT | NT | NT | NT | NT | NT | NT | NA | None | ND | NT | NT | NT | NT | NT | NT | NT | NT | NT |
| MGHK 099 | 12.2 | 19.9 | D | 8.5E-01 | ND | ND | ND | 19.2 | 17.6 | ND | O3b | ND | ND | < 5 | None | D | NEG | NT | POS | NEG | O3 | ND | ND | NT | NT |
| MGHK 100 | 13.1 | ND | ND | ND | NT | NT | NT | NT | NT | NT | NT | NT | NT | NA | None | ND | NT | NT | NT | NT | NT | NT | NT | NT | NT |
| MGHK 101 | 10.7 | 18.2 | D | 9.3E-01 | ND | ND | ND | ND | ND | ND | not typed | ND | ND | > 5 | possible non-typable Kp present | D | NEG | NT | NEG | NEG | not typed | ND | ND | O9 | NT |
| MGHK 102 | 11.2 | ND | ND | ND | NT | NT | NT | NT | NT | NT | NT | NT | NT | NA | None | ND | NT | NT | NT | NT | NT | NT | NT | NT | NT |
| MGHK 103 | 15.6 | ND | ND | ND | NT | NT | NT | NT | NT | NT | NT | NT | NT | NA | None | ND | NT | NT | NT | NT | NT | NT | NT | NT | NT |
| MGHK 104 | 13.6 | ND | ND | ND | NT | NT | NT | NT | NT | NT | NT | NT | NT | NA | None | ND | NT | NT | NT | NT | NT | NT | NT | NT | NT |
| MGHK 105 | ND | ND | PCRI | ND | NT | NT | NT | NT | NT | NT | NT | NT | NT | NA | None | D | NEG | NT | POS | NEG | O3 | ND | ND | NT | NT |
| MGHK 106 | 14.7 | 27.6 | D | 2.2E-02 | 29.2 | 28.8 | 28.4 | ND | ND | 30.9 | O1v2 | O5 | ND | < 5 | None | D | POS | POS | NEG | POS | O1 | O5 | ND | NT | NT |
| MGHK 107 | 14.5 | ND | ND | ND | NT | NT | NT | NT | NT | NT | NT | NT | NT | NA | None | ND | NT | NT | NT | NT | NT | NT | NT | NT | NT |
| MGHK 108 | 14.1 | 33.6 | D | 2.3E-04 | 32.6 | 32.5 | 31.3 | ND | ND | ND | O1v2 | ND | ND | < 5 | None | ND | NT | NT | NT | NT | NT | NT | NT | NT | NT |
| MGHK 109 | 13.4 | 17.1 | D | 1.3E+01 | 14.7 | ND | 15.3 | ND | ND | ND | O2v2 | ND | ND | < 5 | None | D | POS | NEG | NEG | NEG | O2 | ND | ND | NT | NT |
| MGHK 110 | 13.8 | 26.3 | D | 3.0E-02 | 28.6 | 29.2 | 28.9 | 26.9 | 25.5 | 35.4 | O3b | O1v2 | O5 | < 5 | None | D | NEG | NT | POS | NEG | O3 | ND | ND | NT | O1 + O3 + O5 |
| MGHK 111 | 11.9 | 29.1 | D | 1.1E-03 | 29.2 | 28.7 | 28.8 | 32.9 | 29.4 | ND | O1v2 | O3b | ND | < 5 | None | D | POS | POS | POS | NEG | O1 | O3 | ND | NT | NT |
| MGHK 112 | 15.8 | 26.6 | D | 9.7E-02 | 27.7 | ND | 27.3 | 27.0 | ND | ND | O2v2 | O3a | ND | < 5 | None | ND | NT | NT | NT | NT | NT | NT | NT | NT | NT |
| MGHK 113 | 14.0 | 35.7 | D | 5.0E-05 | ND | ND | ND | ND | ND | ND | Not typed | ND | ND | ND | None | ND | NT | NT | NT | NT | NT | NT | NT | NT | NT |
| MGHK 114 | ND | ND | PCRI | ND | NT | NT | NT | NT | NT | NT | NT | NT | NT | NA | None | D | POS | POS | NEG | NEG | O1 | ND | ND | NT | NT |
| MGHK 115 | 12.1 | 37.1 | > CO | 4.9E-06 | NT | NT | NT | NT | NT | NT | NT | NT | NT | NA | None | ND | NT | NT | NT | NT | NT | NT | NT | NT | NT |
| MGHK 116 | 12.6 | 22.5 | D | 1.3E+00 | ND | ND | ND | ND | ND | 22.3 | O5 | ND | ND | < 5 | None | D | NEG | NT | NEG | POS | O5 | ND | ND | NT | NT |
| MGHK 117 | 12.0 | 37.3 | > CO | 4.4E-06 | NT | NT | NT | NT | NT | NT | NT | NT | NT | NA | None | ND | NT | NT | NT | NT | NT | NT | NT | NT | NT |
| MGHK 118 | 16.4 | 32.9 | D | 1.8E-03 | 32.2 | ND | 31.2 | ND | ND | ND | O2v2 | ND | ND | < 5 | None | D | NEG | NT | NEG | NEG | O2 | ND | ND | NT | NT |
| MGHK 119 | 14.7 | 18.7 | D | 1.0E+01 | 30.9 | 30.3 | 30.1 | ND | ND | ND | O1v2 | ND | ND | 11.4 | possible non-typable Kp present | D | NEG | NT | NEG | NEG | not typed | ND | ND | NEG | O1 |
| MGHK 120 | 13.5 | 36.0 | D | 2.9E-05 | ND | ND | ND | ND | ND | ND | Not typed | ND | ND | ND | None | ND | NT | NT | NT | NT | NT | NT | NT | NT | NT |
| MGHK 121 | 11.3 | 35.6 | D | 8.2E-06 | 32.5 | ND | ND | ND | 34.7 | ND | O2a | O3b | ND | < 5 | None | D | POS | NEG | POS | NEG | O2 | O3 | ND | NT | NT |
| MGHK 122 | 13.6 | 19.2 | D | 3.6E+00 | 16.9 | ND | 17.5 | ND | ND | ND | O2v2 | ND | ND | < 5 | None | D | POS | NEG | NEG | NEG | O2 | ND | ND | NT | NT |
| MGHK 123 | 12.0 | ND | ND | ND | NT | NT | NT | NT | NT | NT | NT | NT | NT | NA | None | ND | NT | NT | NT | NT | NT | NT | NT | NT | NT |
| MGHK 124 | 12.1 | 22.9 | D | 9.8E-02 | ND | ND | ND | ND | ND | 22.4 | O5 | ND | ND | < 5 | None | D | NEG | NT | NEG | POS | O5 | ND | ND | NT | NT |
| MGHK 125 | 16.0 | ND | ND | ND | NT | NT | NT | NT | NT | NT | NT | NT | NT | NA | None | ND | NT | NT | NT | NT | NT | NT | NT | NT | NT |
| MGHK 126 | 14.2 | ND | ND | ND | NT | NT | NT | NT | NT | NT | NT | NT | NT | NA | None | ND | NT | NT | NT | NT | NT | NT | NT | NT | NT |
| MGHK 127 | 10.0 | 32.8 | D | 2.4E-05 | 23.3 | 22.3 | 24.2 | ND | 27.6 | 32.0 | O1v2 | O3b | O5 | < 5 | None | D | POS | POS | POS | NEG | O1 | O3 | ND | NT | O1 + O3 + O5 |
| MGHK 128 | 10.8 | ND | ND | ND | NT | NT | NT | NT | NT | NT | NT | NT | NT | NA | None | ND | NT | NT | NT | NT | NT | NT | NT | NT | NT |
| MGHK 129 | 11.6 | 39.6 | > CO | 6.0E-07 | NT | NT | NT | NT | NT | NT | NT | NT | NT | NA | None | D | POS | POS | NEG | NEG | O1 | ND | ND | NT | NT |
| MGHK 130 | 26.8 | ND | ND | ND | NT | NT | NT | NT | NT | NT | NT | NT | NT | NA | None | ND | NT | NT | NT | NT | NT | NT | NT | NT | NT |
| MGHK 131 | 11.5 | 36.2 | > CO | 6.4E-06 | NT | NT | NT | NT | NT | NT | NT | NT | NT | NA | None | D | POS | NEG | NEG | NEG | O2 | ND | ND | NT | NT |
| MGHK 132 | 10.7 | ND | ND | ND | NT | NT | NT | NT | NT | NT | NT | NT | NT | NA | None | D | POS | POS | NEG | POS | O1 | O5 | ND | NT | NT |
